# Characterization of an in vitro 3D intestinal organoid model by using massive RNAseq-based transcriptome profiling

**DOI:** 10.1038/s41598-021-96321-8

**Published:** 2021-08-17

**Authors:** Jing Lu, Anna Krepelova, Seyed Mohammad Mahdi Rasa, Francesco Annunziata, Olena Husak, Lisa Adam, Suneetha Nunna, Francesco Neri

**Affiliations:** Leibniz Institute on Aging, Fritz Lipmann Institute (FLI), Jena, Germany

**Keywords:** Cell signalling, Gastrointestinal models

## Abstract

Organoids culture provides unique opportunities to study human diseases and to complement animal models. Several organs and tissues can be in vitro cultured in 3D structures resembling in vivo tissue organization. Organoids culture contains most of the cell types of the original tissue and are maintained by growth factors mimicking the in vivo state. However, the system is yet not fully understood, and specific in vivo features especially those driven by cell-extrinsic factors may be lost in culture. Here we show a comprehensive transcriptome-wide characterization of mouse gut organoids derived from different intestinal compartments and from mice of different gender and age. RNA-seq analysis showed that the in vitro culture strongly influences the global transcriptome of the intestinal epithelial cells (~ 60% of the total variance). Several compartment-, age- and gender-related transcriptome features are lost after culturing indicating that they are driven by niche or systemic factors. However, certain intrinsic transcriptional programs, for example, some compartment-related features and a minority of gender- and aging- related features are maintained in vitro which suggested possibilities for these features to be studied in this system. Moreover, our study provides knowledge about the cell-extrinsic or cell-intrinsic origin of intestinal epithelial transcriptional programs. We anticipated that our characterization of this in vitro system is an important reference for scientists and clinicians using intestinal organoids as a research model.

## Introduction

Organoid technology, as one kind of 3D culture technology has been widely used in modern scientific researches since the first establishment of long-term 3D culture of intestine organoids from Lgr5 + stem cells was introduced by Sato et al. in 2009^1^. After that, some research groups also used organoids as an in vitro model for screening effective compounds from in-vitro experiments. Ley et al. ever observed that some compounds’ protective effect on irradiation got diminished after several passages of organoids and showed that the organoids’ intrinsic features changed in the process of organoids’ passaging^2^. Further improvements of the cell culture systems have been made to provide in vitro models derived from primary cells of different tissues and that could resemble, as much as possible, the tissue of origin. Various organoid systems were developed to represent the human characteristics of different 3D tissue structures and were applied to study developmental biology, to address human pathological or physiological process, to advance cell therapy and to screen personalized drugs^3^. For instance, brain organoids helped to illustrate the details about brain development and human brain circuitry assembly^4–6^ as well as to model microcephaly^7^, a disorder that has been difficult to recapitulate in mice. It has also been observed that transplantation of human small intestine organoids into immunodeficient mice could yield human epithelial and laminate human mesenchyme, supported by mouse vasculature and it was proved that the transplanted tissue was functional in permeability and peptide uptake tests^8^. However, the utility of this human-modelling in vitro system relies implicitly on the robustness and transferability of the protocol. In addition, some organoids require complicated long stepwise protocols to acquire the desired cell type composition or structure^3^. Unlike methods that generate a relatively homogeneous cell type, the complexity of organoids increases the likelihood of substantial variation of itself. A recent study from Kadosh et al.^9^ showed that the differential p53 response in different intestinal compartments disappeared after organoids culture since it was mediated by the interaction with the gut microbiome. It is tempting to speculate that external and microenvironmental factors as well as intrinsic (epi)genetic background should be rigorously considered when evaluating the robustness of the organoid model. A better understanding of organoids is a prerequisite to improve this model system and make it an exceptional tool for further researches and applications. Here, we provide a comprehensive transcriptional evaluation with small intestine organoids of mice, integrating multiple different variables. Applying RNA sequencing (RNA-seq) to in vivo crypts and in vitro organoids on different compartments, genders and ages, we present how organoids represent the intrinsic and extrinsic characteristics with a comparison of their corresponding in vivo crypts. From our data, we identified a remarkably impressive difference arising between in vivo and in vitro conditions. Deconvolution of cell-type compositions indicates that more stem cells are enriched in culture at the expense of differentiated cells. Some compartment-related transcriptional features in vivo are kept after culture, while most of the aging- and gender-related features are lost. In this study, we demonstrated the limitation of the organoid model in representing in vivo characteristics of the intestinal epithelium. In biology-modelling studies, it is crucial to consider the variation of the culture system, which can impair the study of the biological mechanisms and the cellular response to external stimuli. Careful experiment design and culture condition modification such as choosing appropriate culture media or essential cofactors may be essential to overcome the challenge of culture bias.

## Results

### In vitro culturing of small intestinal crypts as organoids does substantially affect their transcriptional program

**Figure 1:**
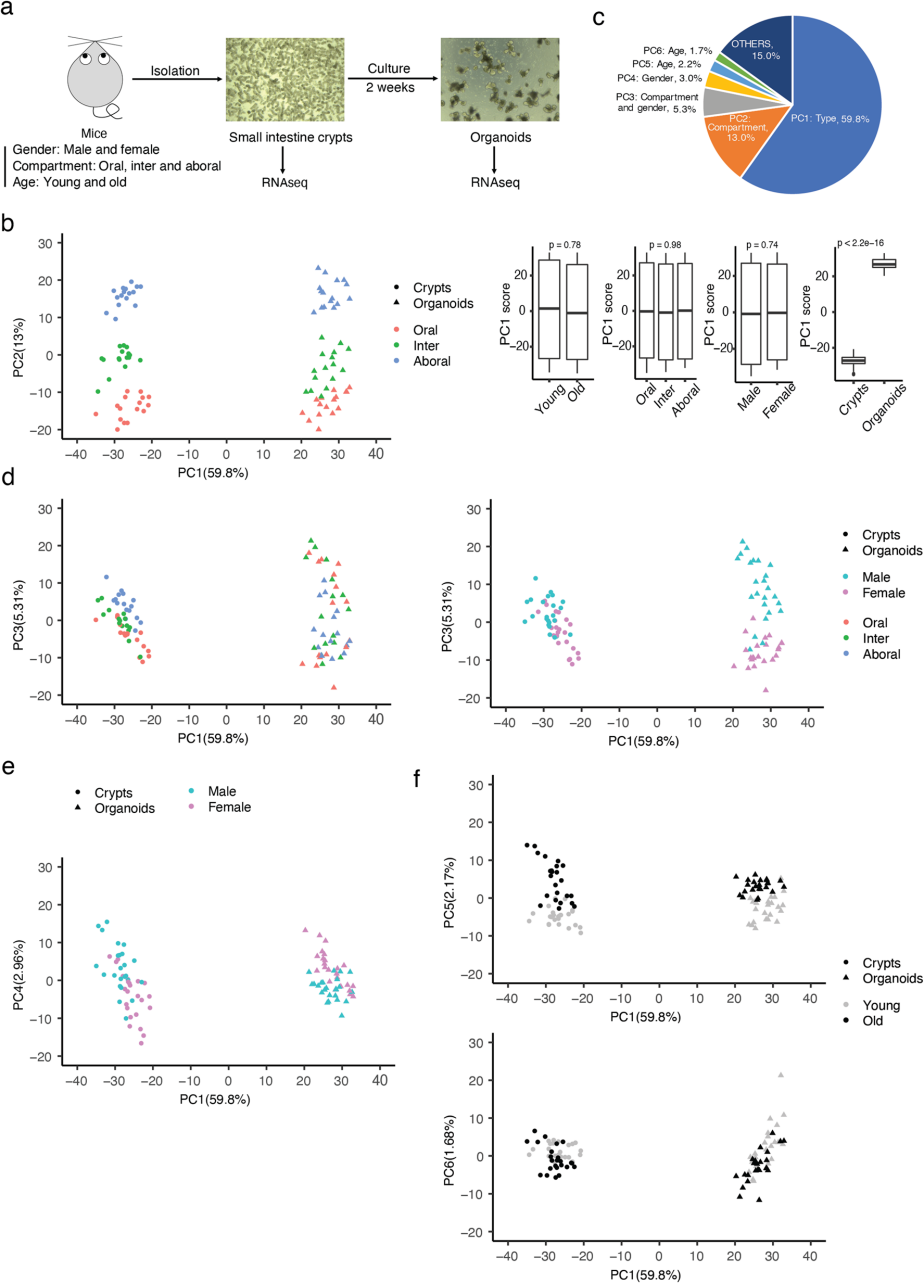
In vitro culturing of intestinal crypts as organoids does substantially affect their transcriptional program. (**a**) Graphic scheme for experiment design. (**b**) Point plot of the first two principal components (left panel) from PCA which were mapped to sample types (crypts and organoids) and compartments (oral, inter, aboral) separately. Boxplot of PC1 versus different biology variables indicated the main difference coming from sample types (right panels). P-value is calculated by Welch’s two-tail t-test. (**c**) Pie chart indicating the variance explanation percentage of top few principal components deconvoluted from meta RNA-seq. (**d**) Point plot of PC3 versus PC1 by mapping PC3 to different groups of compartments (top panel) and gender (bottom panel). (**e**) Point plot of PC4 versus PC1 by mapping PC4 to different groups of gender. (**f**) Point plot of PC5 (left panel) and PC6 (right panel) versus PC1 by mapping PC5/PC6 to different groups of age. See also Supplementary Figs. S1–S3.

In this study, compartment, gender and age were analysed in vivo and in vitro as biological parameters to evaluate the intestinal organoid model. We isolated crypts from the small intestine of male and female, young (10–15 weeks old) and old (85–115 weeks old) wild-type C57BL6/J mice. Crypts were isolated from the oral (duodenum), intermediate (jejunum) and aboral (ileum) compartment of the small intestine. Four biological replicated were used for each condition that resulted in 96 final samples. Crypts were split in two halves and one half was frozen in Trizol for later RNA extraction (in vivo condition), while the other plated on Matrigel to generate organoids. Organoids were collected after two weeks of culturing for RNA extraction. RNA was extracted and sequenced by employing polyA-enriched RNAseq technique (Fig. 1a).

Principal components analysis (PCA) with normalized counts from the RNA-seq data showed a clear separation of the samples. PC1, that could explain 59.8% of variance in our data, displayed the differences between in vivo and in vitro (Fig. 1b,c). PC2, representing 13% of variance, showed compartment-related transcriptional differences (Fig. 1b,c and Supplementary Fig. S1a). Interestingly, these differences are maintained in vitro. The third component (PC3, 5.3% of total variance—Fig. 1d) was deconvoluted for the differences in compartment (left panel) and gender, with the compartment-related feature lost after culture, while the gender-related feature enhanced after culture (Fig. 1d and Supplementary Fig. S1b). PC4 showed another gender-related feature lost (almost reversed) after culture (Fig. 1e and Supplementary Fig. S1c). PC5 (and to a minor extent for PC6) showed minor aging-related features that were partially maintained in vitro (Fig. 1f and Supplementary Fig. S1d,e). Highly contributing genes were defined by the cutoff (− 3.32) of log2 (contribution on principal component) (Fig. 1f). Number of highly contributing genes was balanced in different principal components with 446 highly contributing genes for PC1 and about 300 for other PCs (Fig. S1g). Expression heatmap of the top 10 highly contributing genes of each principal component highlighted the behavior of the different biological parameters between in vitro and in vivo conditions (Supplementary Fig. S1h and Table S1). Highly-contributing genes to PC1 (in vitro vs in vivo difference) were enriched in biological processes like immune response, cholesterol metabolic process and lipid metabolic process (Supplementary Fig. S2a and Table S1, summary in Table 1). Enrichment of immune response pathways such as antigen processing, antigen presentation and defense response underlines the importance of the immune cells in regulating the intestinal epithelial transcriptome^10^. Cholesterol and lipid metabolic processes categorized by PC1 may reflect the need of biomass required in a highly proliferating and dynamic system as the organoids. Adequate availability of building blocks (including lipids) is a prerequisite for cellular proliferation. Wang et al.^11^ ever presented that intestine stem cell proliferation in vivo was promoted by stimulating cholesterol biosynthesis or driving endogenous cholesterol synthesis. He also observed that increasing cholesterol content stimulated organoids’ growth, that strongly supports our observations. Highly-contributing genes to PC2 were enriched in different metabolic processes (e.g. retinoid and glutathione) and transmembrane transport pathway (Supplementary Fig. S2b and Table S1). Different physiological environments along the intestine including different food and microbiota may determine the different metabolic abilities along the compartments. Absorption of some molecules (calcium^12^, small peptite^13^) depends on compartment-specific enterocytes and require particular transmembrane transporters. These PC2-related compartment-associated features arose mostly from differences in the absorption properties of the different intestinal tracts, and remained intact in in vitro culturing, suggesting that these intestinal local functions are driven by cell intrinsic factors, supposedly by development-specific epigenetic programs. Gender-related features (PC3 and PC4) were prone to be reverted in culture (Supplementary Fig. S2c,d and Table S1). After culture, some gender features were enhanced (cell proliferation regulation, cellular response to hypoxia) and some got lost (phospholipid efflux, oxidation process) therefore implying that some of the differences observed between the in vivo and in vitro were gender-biased. Aging features addressed by PC5 and PC6 were enriched in immune system processes, cellular response and ion transport (Supplementary Fig. S2e,f and Table S1).

**Table 1:** Summary of PC1–PC6 on the indicated biology meaning, characteristics and biology processes enriched significantly from highly-contributing genes of corresponding principal components.

PCs	Biology meaning	Characteristics	Significantly enriched biology processes: GO_BP
PC1	General difference between in vivo and in vitro	Main culture difference	Immune system and immune response Cholesterol biosynthetic process Cholesterol metabolic process Lipid metabolic process Antigen processing and presentation Defence response
PC2	Features determining compartment’s specification	Kept after culture	Metabolic processes Retinoid metabolic process Positive regulation of triglyceride catabolic process Transmembrane transport Regulation of intracellular PH Cholesterol homeostasis
PC3	Features promoting the compartments and regulating the sex differences	Sex-enhanced and compartment-lost features after culture	Regulation of blood vessel size Positive regulation of (cell proliferation/toll-like receptor/mitotic nuclear division/phosphatidylinositol 3-kinase) Cellular response to hypoxia Glycolytic process Cholesterol homeostasis
PC4	Features regulating the sex-specific responses	Sex specific features but working in an opposite way after culture	Lipid metabolic processes Phospholipid efflux Cholesterol homeostasis Response to nutrient Triglyceride homeostasis
PC5	Main features determining aging differences (smoothly reduced in culture)	Aging-specific features which were kept after culture	Immune system process Cell adhesion Antigen processing and presentation Cellular response
PC6	Minor features determining aging differences (smoothly increased in culture)	Aging-specific complex features which have various aging patterns	Sodium ion transport Embryonic skeletal system morphogenesis Integrin-mediated signalling pathway Ion transport Regulation of intracellular PH Lymphocyte homeostasis

Gene set enrichment analysis (GSEA) on PC1-related pathways showed that immune system processes were downregulated in organoids (probably because of the lack of immune cells), while cholesterol biosynthesis pathway was upregulated in vitro (Supplementary Fig. S3a and Table S1). Retinol metabolic process and oxidation reduction process (PC2) were enriched in oral part of small intestine both in vivo and in vitro (Supplementary Fig. S3b and Table S1). Interestingly, immune-related pathways in aging (PC5) were upregulated in old crypts, but decreased their enrichment after culture (Supplementary Fig. S3c and Table S1) indicating a cell-extrinsic origin of the factors driving these dysregulations in aging.

All together these results indicate that in vitro culturing strongly affects the transcriptional profile of intestinal epithelial cells, especially for genes whose transcriptional regulation is driven by cell-extrinsic factors as for example genes belonging to the immune response or metabolic biosynthesis pathways. However, intestinal organoids conserved some transcriptional differences coming mainly from diverse compartments, but also, to minor extent, from gender and age suggesting that cell-intrinsic factors, probably sustained by specific epigenetic programs^14^, can regulate these condition-specific gene pathways regulation.

## Cell population analysis revealed higher proportion of stem/TA cells in vitro than in vivo

**Figure 2:**
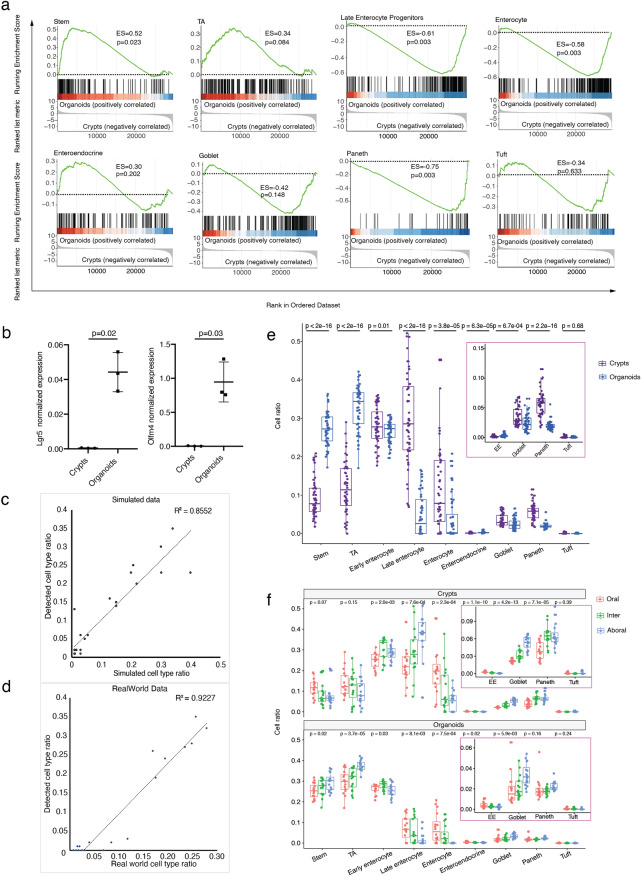
Cell population composition in all the conditions illustrated higher proportion of stem/TA cells in vitro than those in vivo. (**a**) Gene set enrichment analysis (GSEA) between crypts and organoids with intestine epithelial cell-type markers (Stem cell, TA cell, Late enterocyte progenitors, Enterocyte, Enteroendocrine, Goblet, Paneth, Tuft). The enrichment score (ES) reflects the degree to which the genes in a gene set are overrepresented at the top or bottom of the entire ranked list of genes. p-value was calculated using permutation test and was adjusted for multiple comparison by method of “BH” (Benjamini–Hochberg correction). (**b**) Quantitative real-time polymerase chain reaction analysis of relative mRNA expression of stem cell markers (Lgr5 and Olfm4). p-value was calculate using paired two tailed t test. (**c**, **d**) Scatter plot of validation of cell type deconvolution method (CIBERSORTX) on bulk RNA-seq data in simulated data (B) and real-world data (C). R^2^ was performed by Pearson correlation. Feature model was trained by single cell RNAseq (scRNAseq). Simulated data was generated by feature model. Cell type composition in real-world data was defined by single cell RNA-seq. (**e**) Box plot of cell type ratio by group of crypts and organoids (EE: Enteroendocrine). p-value was calculated by Welch’s two-tail t-test. (**f**) Box plot of cell type ratio by group of compartment (EE: Enteroendocrine). p-value was calculated by one-way anova test.

By conducting GSEA with typical gene markers from epithelial cell types, we found that markers of undifferentiated cell types (stem cell and TA cell) were dominantly enriched in organoids, while markers of differentiated cell types (either enterocyte or secretory lineage) were enriched in crypts (Fig. 2a and Table S2). Previous research^15^ already compared the cell composition in long-term cultured intestinal organoids by measuring relative mRNA expression of differentiated cells’ markers (Muc2 for goblet cells, ChgA for enteroendocrine cells, Alp for enterocytes, and Lyz1 for Paneth cells). It was observed that the expression of these markers in organoids was significantly less than in crypts, consistently with our observations. In addition, we performed the validation of two ISC markers (Lgr5 and Olfm4) and we found that the expression of these two markers was elevated pronouncedly after long-term culture (Fig. 2b). This suggested that organoids have different cell population composition from in vivo crypts, resulting in a less differentiated state in vitro than in vivo. We tried to deconvolute cell population in each condition with CIBERSORTx^16^ based on the composition feature model trained from cell type transcriptome profile of single cell data. This cell population deconvolution method was validated to be robust to predict cell population composition from bulk RNA-seq data. We demonstrated that detected cell-type composition in this method had a high correlation with actual cell-type composition in both simulated data (R^2^ = 0.86, Fig. 2c and Supplementary Table S2) and real-world data (R^2^ = 0.92, Fig. 2d and Supplementary Table S2) where the actual cell type composition in real-world data was identified by single cell RNA sequencing (Supplementary Table S2). Cell population of stem and TA increased pronouncedly in vitro in contrast with in vivo, which is consistent with undifferentiated cells enriched eminently in organoids from cell-type GSEA results. Meanwhile, organoids comprised much less population of late enterocyte progenitor, enterocyte, goblet and Paneth than those in vivo (Fig. 2e and Supplementary Table S2). It specified in details that cell population composition had changed notably from in vivo to in vitro. The cell composition in organoids is probably the result of the specific culture conditions that may favor expansion of stem and TA cells at the expense of the differentiated cells. However, since appropriate composition of different cell types and their homeostasis in vivo makes intestinal epithelium play its role normally (absorb nutrients, respond to microbes, function as barrier and help to coordinate immune response)^10^, the shift in cell population composition in vitro may be relevant for functional experiments.

Contrasting intestinal environments (intestinal lumen and lamina propria) along intestinal compartments, have been shown to modulate local cell composition of the intestinal epithelium in order to perform different absorptive, secretory, and antigen- or immune- responding functions^17,18^. Interestingly, our data showed that enterocyte cells decreased, while goblet and Paneth cells increased from oral to aboral compartment and these features were maintained in vitro (Fig. 2f and Table S2). In contrast, the oral-to-aboral ratio had an inverted trend in regard to Stem, TA and late enterocyte progenitors between in vivo and in vitro conditions (Fig. 2f). This suggests that compartment-associated cell composition differences may be driven by both cell intrinsic factors and external environmental influences.

### Meta-analysis of biological features (compartment, gender and aging) revealed how biology features changed in culture

**Figure 3:**
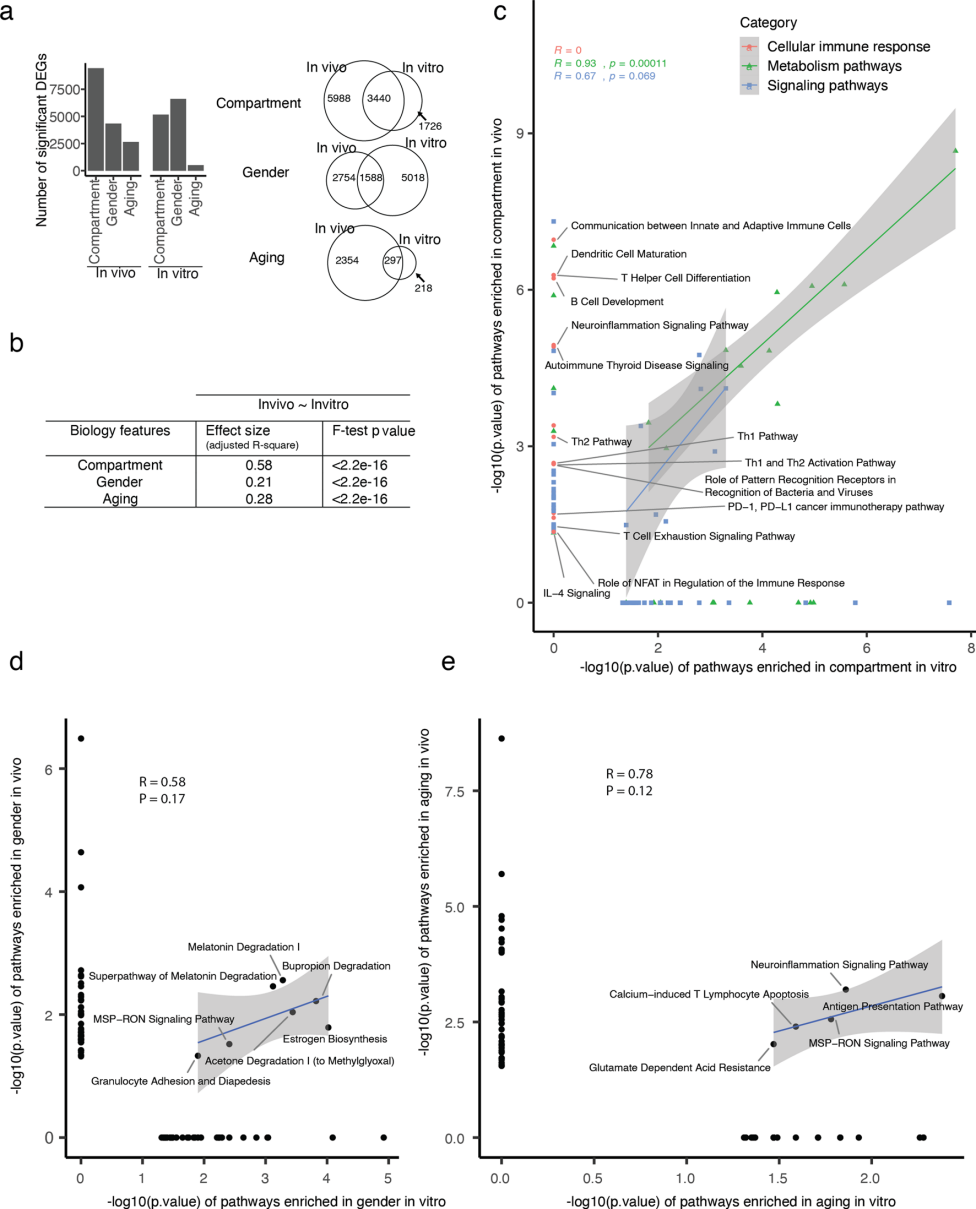
Meta-analysis on specific biology features (compartment, gender, aging) respectively revealed how biology features changed in culture. (**a**) Bar charts indicating the number of significantly differentially expressed genes (DEGs) found in different conditions (compartment, gender, aging) from in vivo and in vitro (left panel). Venn diagrams of the DEGs in the indicated conditions as in the left panel between in vivo and in vitro. (**b**) Table of effect size of in vitro representing in vivo defined by simple linear regression model. Effect size was defined by the adjusted coefficient of determination (adjusted R^2^) of the linear model. F-test p-value was used to evaluate the linear model on our data. (**c**–**e**) Scatter plot between in vivo and in vitro on the enriched pathways in different conditions: compartment (**c**), gender (**d**) and aging (**e**), indicating most biology features got lost after culture. Pathways clustered together were summarized in super categories. Coefficient (R) and p-value were calculated by method of “pearson” correlation. See also Supplementary Figs. S4–S8.

The fluctuation of cell population composition in culture may initiate some further functional changes in the organoids, making the in vitro model a different system from in vivo. In this perspective, it required a further detailed functional investigation on the organoids in comparison with crypts. In this study, we proceeded meta-analysis to compare the pathways between in vivo and in vitro in the views of compartment, gender and aging. Differentially expressed genes (DEGs) were defined for each biological feature in a way of meta-analysis (Fig. 3a) or individual analysis (Supplementary Fig. S4a). In vivo, we found compartment had the most DEGs, and overlapped the most between in vivo and in vitro (Fig. 3a, Supplementary Fig. S4b and Table S3) confirming the results from Fig. 1 where PC2-associated compartment-driven variance was maintained between in vivo and in vitro conditions. With respect to compartment and aging, more in vitro specific DEGs were identified when comparing male vs female mice especially if old (Fig. 3a, Supplementary Fig. S4c and Table S3). This can explain the gender features in PCA analysis (Fig. 1d,e), which identified two main gender-related principal components (PC3 and PC4). Among these two principle components, PC4 represented a reversed gender feature between in vivo and in vitro. DEGs during aging in crypts are the least compared to compartment and gender and are even fewer when we calculated them in organoids (Fig. 3a, Supplementary Fig. S4d and Table S3) indicating that aging-induced transcriptional alterations are mainly lost when intestinal epithelial cells are taken out from the in vivo environment.

Linear model analysis on DEGs showed in vitro compartment features represented 58% to those in vivo, while it is 21% in gender and 28% in aging (Fig. 3b). The least effect size for gender in culture could be explained by the most in vitro specific gender features. Some transcriptional features associated respectively with compartment, aging and gender were still kept after culture, with compartment had the most features kept after two weeks culturing, followed by aging and finally gender. Functional enrichment analysis demonstrated that compartment differences in vivo were enriched in cellular immune response, signaling pathways, and metabolism pathways (Supplementary Fig. S5a and Supplementary Table S3). After culture, most pathways of compartment-related cellular immune response got lost (Fig. 3c, red dots), while signaling and metabolism pathways were kept with a similar enrichment (Fig. 3c, blue and green dots, Supplementary Fig. S5b and Table S3). These data suggest that cellular immune response pathways like T-helper or B-cells related pathways could be working differently between oral and aboral parts because of interaction with different bacterial or viral antigens in different compartments and these interactions are lost in the culture condition.

Gender and aging related differences were rather changing upon culturing. Gender differences in vivo were enriched in cellular immune response (e.g. T helper related pathways, antigen presentation pathways, antiviral response) and signaling pathways (Supplementary Fig. S6a and Table S3). Gender-related pathways related to metabolism pathways, cytokine-related signaling pathways (Interleukin signaling, NF-kB signaling) and MAPK signaling dramatically changed after culture (Fig. 3d, Supplementary Fig. S6b and Table S3). Aging differences in vivo were enriched in cellular immune response (antigen presentation pathways, T-cell related and B-cell related pathways), amino acid metabolism (glutamate degradation, glycine betaine degradation, methionine salvage II) and immune signaling response (interleukin signaling, interferon induction, HMGB1 signaling) (Supplementary Fig. S7a and Table S3). After culture, only some of these in vivo aging pathways (MSP-RON signaling pathways, antigen presentation pathway) were partially kept in vitro (Fig. 3e, Supplementary Fig. S7b and Table S3). Antigen presentation pathway was still found significantly enriched between young and old suggesting that the culture period of 2 weeks may be not enough to abolish aging influence on this pathway. Taken together, only few in vivo pathways of gender and aging could be kept in culture (Fig. 3d,e) indicating a limited potential of organoids in representing gender and aging features in long term cultures.

To further investigate the difference of upstream regulators inducing these pathways, some pronounced regulators were ranked by significance (Supplementary Fig. S8 and Table S3). AGT (Angiotensinogen) and beta-estradiol were predicted to be the upstream regulator for compartment differences both in vivo and in vitro even-though with a different enrichment p value (Supplementary Fig. S8 and Table S3). TGFB1, TNF and IL6 were responsible for compartment differences in vivo*,* while CDX genes became a dominant regulator in vitro. In gender, we found many regulators (FYN, TNF, TLR7, PRKD1) affecting gender differences in vivo while only few significant ones could be detected in vitro. IFNG was detected as general regulator of aging both in vivo and in vitro. IL12 and TNF were also playing promising roles in influencing aging in vivo.

### Characterization of organoids as an in vitro model for aging research

**Figure 4:**
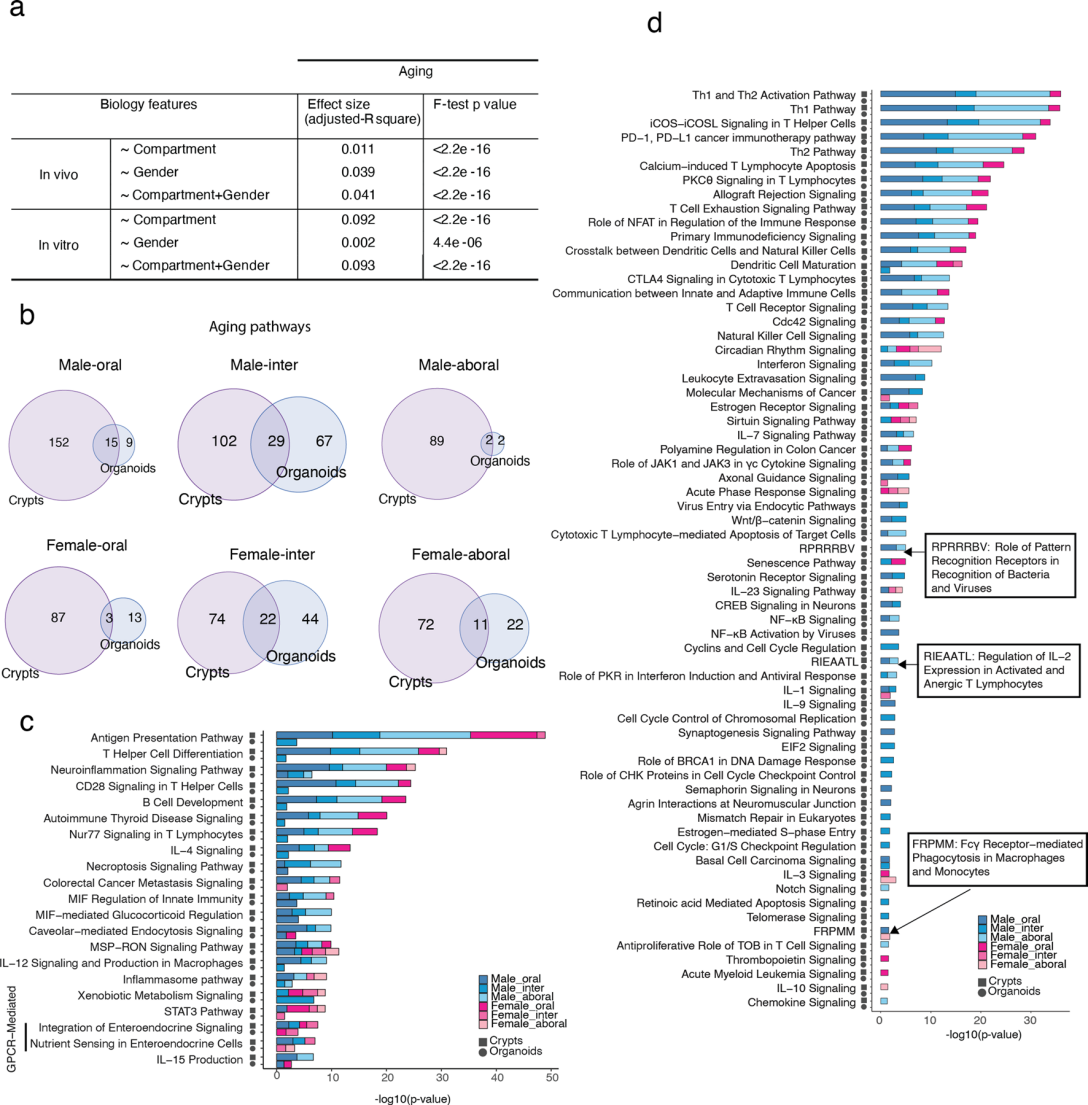
Characterization of organoids as an in vitro model for aging research. (**a**) Table of effect size of compartment, gender or (compartment + gender) on aging in vivo or in vitro by linear regression model. Effect size was defined by the adjusted coefficient of determination (adjusted R^2^) of the linear model. F-test p-value was used to evaluate the linear model on our data. (**b**) Venn diagrams of the significantly enriched pathways during aging in different conditions between in vivo and in vitro. (**c**) Stacked bar chart of − log10 enrichment p-value (fisher exact test) in some typical relevant pathways found dysregulated in aging in vivo, which were kept in culture in at least one condition. (**d**) Stacked bar chart of − log10 enrichment p-value (fisher exact test) in some typical relevant pathways found dysregulated in aging in vivo, which were lost in culture. See also Supplementary Fig. S9.

To see how much compartment and gender had an impact on aging, we disentangled the compartment and gender effects on the aging-induced transcriptome alteration. Differentially expressed genes (DEG, p value < 0.05) coming from all the pairwise comparisons young vs old were used to construct a matrix with log2-fold-change as values. Complex differences of biological features by DEG matrix were illustrated by linear model (Fig. 4a). The effect of gender on aging in vivo was higher (3.9%) than that of compartment (1.1%), while it almost disappeared (0.2%) in vitro differently from the compartment effect on aging that were enhanced by culturing. This could be caused by the strong differences observed in gender features after culture. The effect of compartment on aging in vitro reached up to 9.2%, that could be disclosed by the shrink of aging features and the maintenance of compartment features in culture.

About 12% of aging-associated pathways were maintained in culture (Fig. 4b and Supplementary Table S4). To specify how aging-induced transcriptional alterations changed after culture, we summarized functional pathways conserved or disappeared upon 14 days of culturing. Few of the aging-related pathways like some of the cellular immune response, intracellular signaling, cellular growth and proliferation, degradation, and metabolic pathways were found enriched also in organoids, even though to a massive lower extent (Fig. 4c, Supplementary Fig. S9a and Table S4). Most of these pathways were statistically enriched in multiple in vivo conditions (e.g. in multiple compartments and/or in both the genders), but in organoids were mostly found only in one condition (Supplementary Fig. S9b). This may indicate that in some conditions (e.g. male-intermediate) the effect of aging in altering the transcriptional landscape may be stronger than in other conditions and, therefore requiring longer culture time to get rescued. The rest of the aging-related pathways completely disappeared upon culturing (Fig. 4d, Supplementary Fig. S9c and Table S4).

All these data further proof that organoids do not represent an optimal research model to study aging-associated intestinal transcriptional phenotypes and that very few aging-related pathways were kept upon culturing. On the other hand, suggest they that old intestinal epithelium has the intrinsic potential to restore young-like transcriptional phenotype.

## Discussion

In this study, we used multi-variable transcriptomics of intestinal tissue to obtain a global and unbiased view of in vitro organoid model on representing in vivo characteristics. We addressed a detailed comparison of the transcriptional profiles of mouse small intestinal crypts freshly isolated or after 2 weeks of culturing as 3D organoids. We used crypts from different small intestinal tracts (duodenum, jejunum and ileum) extracted from mice of different gender and age. Culture explained the largest variance (almost 60%) among all our biological variables, that was explained by the adaptation to the new environment and by a change in cell-type composition. In particular, transcriptional programs associated with immune system were lost indicating that the immune cells of the lamina propria play an important role in communicating with the epithelial cells of the intestine. These interactions are lost in organoid culturing suggesting that co-culturing of intestinal organoids with immune cells may be of extreme relevance as pointed out by the fact that these strategies have been already implemented in some research areas like cancer research^19^. Secondly, we observed that metabolic pathways were differently enriched between crypts and organoids, especially lipid metabolism suggesting that carbon and energy sources may be different between in vitro and in vivo and that the cells may have also differences in cell proliferation. These data strongly suggest that the culture conditions should be improved with the aim to reach conditions as more similar as possible to the in vivo situation. Finally, our analysis indicated organoids had a cell composition of more undifferentiated cell types than in vivo, which may reflect the optimal conditions for growth that are established in vitro.

Thanks to the multiple variables integrated in our design, meta-analysis of the complex features allowed us to achieve an evaluation of in vitro model with unbiased results. By applying linear model for different biological variables, we were able to disentangle different biological features and to interpret how the complex biological features change after culture. We found only a proportion of biological features could be maintained after culture. Compartment-associated differences were the most stable in culture indicating that they can be driven by cell-intrinsic factors (like epigenetic programs establish during development), however only a little more than half of the differences was kept upon 2 weeks of culturing. This can explain why compartment-specific response to p53 mutation disappeared in culture in Kadosh’s research^9^. Gender-related features were almost completed lost in culture suggesting that they are almost exclusively mediated by cell-extrinsic (probably systemic, like hormones) factors in vivo. Interestingly, in some cases, the culture reversed the gender-associated transcriptional differences observed in crypts.

With a relatively small fraction of common aging features (28%) observed both in vitro and in vivo, organoids as an aging model need to be further investigated with caution. In this study, we showed that differentially expressed genes in young vs old samples decreased greatly after culture especially in oral and aboral compartment. These data indicate that the aging phenotypes of an aged intestine may be derived from the deterioration of the intestinal microenvironment (niche cells, immune system of the *lamina propria*) and by alteration of systemic factors like diet, microbiome or organismal factors. Aging is characterized by a constitutive pro-inflammatory environment with persistent low-grade innate immune activation that may augment tissue damage caused by infections in elderly individuals^20^. Absence of immunosenescence in vitro can limit organoids as well. Some other aging pathways occurring in certain conditions, like cytokine-related signaling (most interleukin signaling pathways, Interferon signaling, NF-κB signaling), cell development (Wnt/β-catenin signaling, senescence pathway, notch signaling, retinoic acid mediated apoptosis signaling), cell cycle (cell cycle regulation, cell cycle control of chromosomal replication, Role of CHK proteins in cell cycle checkpoint control, estrogen-mediated S-phase entry, cell cycle: G1/S checkpoint regulation), DNA repair (role of BRCA1 in DNA damage response, mismatch repair in eukaryotes), neuron interaction (CREB signaling in neurons, synaptogenesis signaling pathway, semaphoring signaling in neurons, agrin interactions at neuromuscular junction), could not be enriched in aging any more after culture (Fig. 4d, Supplementary Fig. S5c and Table S4). Lack of interaction with nervous system in vitro made aging specific pathways like GNRH signaling^21^, opioid signaling pathway^22^, CREB signaling in neurons^23^ not able to be present in vitro, which also restricted organoids in the application of aging research. In spite of these limitations, some aging functional pathways were observed in vitro which could be referred to some investigation on specific aging functions. Xenobiotic metabolism has been proposed to play a role in modulating the rate of aging. Many phase I xenobiotic metabolizing enzymes genes are upregulated in long-lived mouse models^24^. Expression levels of JAK-STAT signaling targets in increased with age, and STAT3^25^ has been implicated in the regulation of self-renewal and stem cell fate in several tissues (Fig. 4c and Supplementary Fig. S5a).

Some of the transcriptional features characteristic of an aged intestine were still present after 2 weeks of culture suggesting that, for some studies, freshly isolated crypts can be used for short-term experiments. For long-term experiments, this study provides the basis to build specific culture conditions of an aged environment for example by supplementing/modulating specific cytokines, metabolites, or growth factors. On the same line, also co-culturing organoids with some certain bacteria species or immune cells has been recently made possible by employing specific novel strategies like the organ on chip technology^26,27^.

Organoids are a rather novel in vitro system widely used. Different from 2D culture, organoid model can better represent the tissue structure/polarization, cell heterogeneity and cell-to-cell communication features. The use of standardized protocols and guideline for the organoid culture is an important issue in order to reduce the variability of the system among different studies^3^. However, it is tempting to speculate that personalized culture conditions should be employed depending on the research field and on the biological questions. In this study, we provide a potential way for a well-defined quality control to evaluate the organoids establishment by using the transcriptome analysis. However, model evaluations including more dimensions (epigenomic or metabolomic level) would make the characterization even more comprehensive and persuasive. Although more expensive, single-cell RNA-seq could provide a more accurate and detailed evaluation especially in the aspect of cell population composition. In this research, we only applied a classic ENR medium (Epidermal growth factor/Noggin/R-spondin1) for organoids establishment. Epidermal growth factor (EGF) signaling is associated with intestinal proliferation^28^. Transgenic expression of Noggin induces expansion of crypt numbers^29^. R-spondin-1 is a ligand for Lgr5, a marker for intestinal stem cells^30^, and an essential factor to activate Wnt signal in intestineal crypts^31^. It is also possible to manipulate the organoids with different medium. By adding CHIR99021 and VPA to the ENR medium, a previous study^32^ showed that it is possible to modulate cell composition in organoids to enrich stem cells by stimulation of Wnt signaling and Notch pathway. As a comparison, another research^15^ revealed that continuous passaging under ENR-CV conditions, but not ENR conditions would induce a phenotypic changes of reduced Lgr5 + cells. The maintenance of the characteristics of intestinal organoids upon extended passage is mediated by ENR condition, but not ENR-CV condition, which indicated the importance of passaging time on modeling organoids. Han et al. ever showed that cell composition was different between organoids cultured in ENR and ENR-CV (ENR/CHIR99021/VPA) with more proliferative cells and less differentiated cells in organoids cultured in ENR-CV especially in early phase (P0-4)^15^. In their research, they also found markers of differentiated epithelial cells (Lyz1, Muc2, ChgA, ALP) expressed less in organoids than raw crypts which is consistent with our findings. Qu et al.^33^ established a novel hyperplastic intestinal organoids (Hyper-organoids) by applying an intestinal organoid culture system composed of 8 components mainly including VPA, EPZ6438, LDN193189, and R-Spondin 1 conditioned medium. VPA and EPZ6438 was revealed to be indispensable for epigenome reprogramming and regeneration in Hyper-organoids by functioning through epigenetically regulating YAP signaling. Furthermore, Boonekamp et al.^34^ had recapitulated that intestinal organoids could be manipulated for enriching different cell types by applying different compounds to the ENR medium to direct development signaling (e.g. Notch, EGF, BMP and Wnt). Addition of the NOTCH inhibitor DAPT to stem cell-enriched murine intestinal organoids promotes secretory cell differentiation but favors beyond what to be expected given the natural abundance of each lineage in vivo, simultaneous addition of DAPT and CHIR (ENR + DC) could promote Paneth cell differentiation^32^. Simultaneous addition of DAPT and the Wnt pathway inhibitor IWP2 (ENR + DI) results in organoid cultures favoring goblet cell differentiation^32^. Our evaluation provided an insight of organoids as an in vitro model and a potential thinking for its modification. A more refined protocol for organoids establishment could be possible by coculturing with other stimuli like cytokines, immune cells, nerve cells, microbes, or even blood serum. It is a perspective for a standard evaluation pipeline to be established and applied on different coculture systems in order to get a superior in vivo-mimicking in vitro model.

## Methods

### Mice

All wild-type C57BL6/J mice were group housed and maintained in a Specific Opportunist Pathogen Free (SOPF) animal facility in Fritz Lipmann Institute with 12 h of light/dark cycle and fed with a standard mouse chow. Experiments were conducted according to protocols approved by the state government of Thuringia—THÜRINGER LANDESAMT FÜR VERBRAUCHERSCHUTZ (licenses number: TG/J-0002858/A; TG/J-0003616/A; TG/J-0003681/A). The study was carried out in compliance with the ARRIVE guidelines.

### Small intestine crypts isolation

Mouse small intestine was dissected, cut into 3 equal parts (the proximal part adjacent to stomach was termed as “oral” region; the middle part was termed as “intermediate” region; the distal part adjacent to cecum was termed as “aboral” region), and flushed with cold PBS several times. Small intestinal crypts were isolated using the established protocol^35^ with some modifications. First, the small intestine was opened longitudinally and villi were removed by scraping with a coverslip. The small intestinal pieces (2 cm) were washed several times in cold PBS and transferred to 5 mM EDTA/PBS, followed by two 30-min incubations at 4 °C on a rotator. Next, the tissue was transferred to 30 ml fresh cold PBS and manually shaken for 30 s. The crypt solution was filtered using a 70 μm cell strainer and centrifuged at 120xg for 5 min at 4 °C. One part of the isolated crypts was immediately resuspended in QIAzol Lysis Reagent (Qiagen) and stored at − 80 °C for RNA isolation. The remaining part of the isolated crypts was cultured as organoids for 2 weeks.

### Organoid culture

Small intestinal organoids were cultured according to the established protocol^1^. Approximately 200 crypts were mixed with 20 μl of Matrigel (Corning) and plated in 24-well plates. After polymerization of Matrigel, 500 μl of crypt culture medium (Advanced DMEM/F12 (Gibco), 1 × Glutamax (Gibco), 10 mM HEPES (Gibco), N2 supplement (1:100) (Gibco), B27 supplement (1:50) (Gibco), 0.5 U/mL penicillin/streptomycin (Gibco), 50 ng/ml mouse recombinant epithelial growth factor (PeproTech), 100 ng/ml mouse recombinant Noggin (PeproTech), and 500 ng/ml human recombinant R-spondin1 (PeproTech)) was added.

### RNA isolation

Total RNA from crypts and organoids was isolated using QIAzol Lysis reagent (Qiagen) followed by isopropanol precipitation. Isolated RNA was quantified on Qubit 3.0 (Thermo Fisher Scientific). The quality of isolated RNA was analyzed by Fragment Analyzer (Agilent).

### Reverse transcription and real-time PCR

iScript cDNA Synthesis Kit (Bio-Rad Laboratories GmbH 1708891) was used for cDNA synthesis starting from RNA according to the manufacture protocol. Relative gene expression analysis was done using quantitative real-time PCR method (qRT-PCR). The expression levels of target genes were normalized to that of the endogenous housekeeping gene EpCam. The gene expression was determined using the relative Ct method:

$$  {\text{Relative }}\;{\text{expression}} = 2^{{ - ({\text{Ct}}\;{\text{ gene }}\;{\text{of }}\;{\text{interest}} - {\text{Ct}}\;{\text{ housekeeping}}\;{\text{ gene}})}}. $$

### RNA-sequencing library preparation

Poly(A) RNA-seq library preparation was performed as described previously^36^ by using the TruSeq RNASample Prep kit (Illumina) following the manufacturer’s instructions.

### Droplet-based scRNA-sequencing

Proximal small intestinal crypts were isolated as described above and resuspended in 1 ml of Single-Cell Isolation Solution (TrypLE supplemented with 1 mg/ml DNaseI, 5 mM MgCl_2_, 80 μM Y27632) and incubated for 20 min at 37 °C with short vortexing after first 10 min of incubation. Reaction was quenched by addition of 29 ml ice-cold PBS and cells were centrifuged at 800xg for 5 min at 4 °C. Cell pellet was resuspended in FSM supplemented with 80 μM Y27632 and treated with CD326 (EpCAM) (G8.8), PE-Cyanine7 coupled rat monoclonal antibody for 30 min on ice in the dark. The cells were then centrifuged at 450xg for 5 min at 4 °C, resuspended in 500 μl fresh FSM and FACS sorted. In order to yield about 4000 cells, 8000 EpCAM + single cells from young and old mice were flow-sorted into a BSA coated tube containing 1.5 μl PBS with 0.04% BSA. Subsequently, the cell suspension was carefully mixed with reverse transcription mix using the Chromium Single Cell 3’ Library & Gel beads chemistry v2 (10 × Genomics) and loaded into a Chromium Single Cell A Chip (10 × Genomics). During the encapsulation process in the 10X Genomics Chromium system^37^, the cells lysed within the droplet and released polyadenylated RNA bound to the barcoded bead that was captured with the cell. Following the guidelines of the 10 × Genomics user manual, the droplets were directly subjected to reverse transcription, the emulsion was broken and cDNA was purified using Dynabeads MyOne Silane (Thermo Fisher Scientific). After the amplification of cDNA with 9 cycles, it underwent purification and a quality control check on the Fragment Analyzer (Agilent). The cDNA was fragmented for five minutes and dA-tailed, followed by an adapter ligation step and an indexing PCR of 10 cycles in order to generate libraries. After quantification, the libraries were sequenced on NextSeq 500 platform (illumina) using a high-output flowcell in PE mode (R1: 26 cycles; I1: 8 cycles; R2: 57 cycles), thus generating ~ 60 000 fragments on average per cell.

### High-throughput sequencing

All the samples were sequenced on the NextSeq500 platform (illumina, San Diego, CA, USA).

### RNA-sequencing data analysis

Fastq files quality check was performed using FastQC v0.11.5. The fastq files were mapped to the mm9 genome using TopHat v2.1.0^38^ with the following parameters –bowtie1 –no-coverage-search -a 5. The number of reads covered by each gene is calculated by HTSeq-Count 0.11.2^39^ with -s no -a 0 -t exon -m intersection-nonempty parameters. Before further analysis, all of the rRNA genes are removed from the count data. For calculating differentially expressed genes and normalized count, DESeq2 R package v1.20.0^40^ was used with the default parameters. To make it interpreted easily, DEG analysis for compartment is proceeded by aboral part with a reference of oral part of the small intestine. For Pearson correlation analysis and plotting the expression, the normalized count was used. For functional and pathway analysis as well as for identification of potential upstream regulators, DESeq2 differentially expression analysis (adjusted p-value < 0.05) results were uploaded in IPA (Ingenuity Pathway Analysis v45868156)^41^. Only the highly predicted or experimentally validated predicted upstream regulators mRNAs were used for downstream analysis. For gene set enrichment analysis, normalized counts (for each gene in all of the samples) were scaled using scale function in R (with center = TRUE, scale = TRUE parameters). The Z-score was calculated by multiplying the scaled counts by + 1 or − 1 that shows the direction of the expression in aging (+ 1 for up-regulated genes and − 1 for down-regulated genes). The average of Z-scores were calculated for each group and used for drawing plot and downstream analysis.

### scRNA-sequencing data analysis

The raw sequencing data was processed with the ‘count’ command of the Cell Ranger software (v2.1.0, 10 × Genomics) with the default options. The required reference was built with the ‘mkref’ command of Cell Ranger based on the murine genome mm10 as well as the gene annotation from Ensembl (v87) as input. The annotation was filtered with the ‘mkgtf’ command of Cell Ranger to include only protein-coding, lincRNA and antisense gene features (‘–attribute = gene_biotype:protein_coding –attribute = gene_biotype:lincRNA –attribute = gene_biotype:antisense’). The count file was directly uploaded into R using cellrangerRkit package v2.0.0. Before further analysis on the count data, non-expressed genes were removed and the counts were normalized to the total number of counts in each cell (using normalize_barcode_sums_to_median function in cellrangerRkit package) and transformed to log 10 for all of the downstream analysis. PCA was calculated using prcomp function with center = TRUE, scale.  = TRUE parameters. Clustering of the cells was done using kmean clustering (center = 9, nstart = 10) and the cell type of each defined cluster was determined using the well-known markers for each cell types. With (using) 9 clusters, Tuft and Enteroendocrine cells were in in the same cluster, so we separated these cell types by re-clustering using kmean (center = 2, nstart = 10). To find/define markers for each cluster we used order_cell_by_clusters and prioritize_top_genes (method = "sseq", min_mean = 0.1) respectively. The expression of markers was compared between the desired cluster versus all other cells (using prioritize_top_genes function) and the genes with log2 fold change ≥ 2 and adjusted p-value < 0.05 were selected as the marker.

### Principal component analysis

PCA input matrix was constructed by selecting highly-variable expressed genes (the interquartile range, IQR > 1.5) with normalized counts > 10 in more than 3 conditions. Principal component values, PC-contributing values, and PC-weighted genes were extracted by built-in R function prcomp(). Highly-weighted genes for each principal component were defined by the cutoff of density distribution. Corresponding enriched biology processes for each principal component were obtained from DAVID website^42,43^.

### GSEA on celltypes enrichment

GSEA was runned by the package of “clusterProfiler”^44–46^ using the default parameters. Cell type markers were defined by our single cell data. The enrichment score (ES) represent the degree to which a set S is over-represented at the top or bottom of the ranked list L. The score is calculated by walking down the list L, increasing a running-sum statistic when encountering a gene in S and decreasing when it is not. The magnitude of the increment depends on the gene statistics. The ES is the maximum deviation from zero encountered in the random walk, that corresponds to a weighted Kolmogorov–Smirnov-like statistic. P-value was calculated using permutation test and was adjusted for multiple comparison by method of “BH” (Benjamini–Hochberg correction).

### GSEA on pathways enrichment

Scaled expression of each gene in the gene set of GO pathways on normalized counts was proceeded by scale() function in R. Mean z-score was calculated in one condition and displayed the boxplot for all genes in each gene set.

### Cell population deconvolution

Cell populations in all conditions were deconvoluted from an analytical tool called CIBERSORTx^47^. Reference of gene expression profiles in each cell type comes from our single cell data. Corresponding bulk RNA were also sequenced to be as the data set of real-world validation. Simulated bulk RNA data were established according to the reference expression profile from each cell type, regarding of the diverse constitution of different cell types.

### Effect size estimations on biology features

To understand to what extent all these biology features are affected from each other, a multiple linear regression or a simple linear model was used, as implemented in the R (lm()). As DEG results were taken to be representing the biology feature, input matrix was gathered by significantly differentially expressed genes (adjusted p value < 0.05) in vivo or in vitro with Log2FoldChange as values, not significantly differentially expressed genes in other conditions were defined as 0. We compared models that predict in vivo features, using as predictors: (i) in vitro compartment, (ii) in vitro gender, (iii) in vitro aging and (iv) the combination of in vitro compartment, gender and aging together. We also did aging as outcome features in vivo (or in vitro), using as predictors: (i) compartment, (ii) gender and (iii) the combination of compartment and gender. The adjusted R2 square derived from linear model is reported as a measure of effect size. We assessed the quality of each model by F-statistic and the associated p-value. This was done for every module considered. Once significant, this means at least one of the predictor variables is significantly related to the outcome variable.

### Statistical analysis

Data represent mean ± SD. Results were considered significant when p < 0.05. Multiple comparisons among three groups were performed by using the ANOVA test. Then the comparisons between two groups were performed using the two-tailed t test. The comparisons in the same individual were performed with paired t test.

## Supplementary Information

Supplementary Information 1.

Supplementary Information 2.

Supplementary Information 3.

Supplementary Information 4.

Supplementary Information 5.

## Data Availability

The accession numbers of all raw sequencing data reported in this paper can be found in the key resource table. Accession number of the Super Series: GSE169368. Custom scripts are available upon request. All methods were performed in accordance with the relevant guidelines and regulations.
