# Supplementary material for: Characterization of an in vitro 3D intestinal organoid model by using massive RNAseq-based transcriptome profiling
Source: Sci Rep. 2021 Aug 17;11:16668. doi: 10.1038/s41598-021-96321-8 (PMC8371140; doi:10.1038/s41598-021-96321-8)
Supplement: Supplementary file 1 — Supplementary Information 1. [file 41598_2021_96321_MOESM1_ESM.pdf]

Title
Characterization of an *in vitro* 3D intestinal organoid model by using massive RNAseq-based transcriptome profiling

Authors/Affiliations
Jing Lu[1], Anna Krepelova[1], Seyed Mohammad Mahdi Rasa[1], Francesco Annunziata[1], Olena Husak[1], Lisa Adam[1], Suneetha Nunna[1], Francesco Neri[1*]

1 Leibniz Institute on Aging, Fritz Lipmann Institute (FLI), Jena, Germany;
* Correspondence: francesco.neri@leibniz-fli.de

Supplementary information

Supplementary Figures

Figure S1. ***In vitro*** **culturing of intestinal crypts as organoids does substantially affect their transcriptional program. Related to Figure1.**
a, b, c, d, e) Boxplots of PC2-PC6 (a-e) shown in different groups by age, compartment and gender separately *in vivo* and *in vitro*. P-value is calculated by Welch's two-tail t-test for age groups and gender groups while one-way anova test for compartment groups. ns: p>=0.05, *: p<0.05, **: p<0.01, ***: p<0.001.
f) Density plot of gene contributions on PC1-PC6. Cutoff of highly contributing genes was defined as log2(contribution on PC) more than -3.32 shown as dashed line in x axis.
g) Bar chart of number of highly contributing genes to PC1-PC6 indicated balanced selection. These genes were used for biology progresses enrichment analysis in Table 1.
h) Heatmap of the expression level (scaled counts) of the top 10 highly-contributing genes for PC1-PC6 (top panel).

Figure S2. ***In vitro*** **culturing of intestinal crypts as organoids does substantially affect their transcriptional program. Related to Figure1.**
a, b, c, d, e, f) Bubble plot of significantly enriched biology processes by highly-contributing genes of PC1-PC6 (a-f). Gene size was defined how many genes in our input were enriched in one function, gene ratio was defined as gene size divided by size of overlap of input genes with database genes' collection. P-value was calculated by Fisher exact test.

Figure S3. ***In vitro*** **culturing of intestinal crypts as organoids does substantially affect their transcriptional program. Related to Figure1.**
a) Box plots of Z-score from gene sets of immune system process (left panel) and cholesterol biosynthesis pathway (right panel) across sample types (crypts and organoids). P-value is calculated by Welch's two-tail t-test. The gene sets used here were drawn from the Molecular Signatures Database (MSigDB).
b) Box plots of Z-score from gene sets of retinol metabolic process (top panel) and oxidation reduction process (bottom panel) across compartments (oral, inter, aboral). P-value was calculated by one-way anova test. The gene sets used here were drawn from the Molecular Signatures Database (MSigDB).

c) Box plots of Z-score from gene sets of immune system process across aging (young and old). P-value was calculated by Welch's two-tail t-test. The gene sets used here were drawn from the Molecular Signatures Database (MSigDB).

Figure S4. Meta-analysis on specific biology features (compartment, gender, aging) respectively revealed how biology features changed in culture. Related to Figure3.
a) Bar charts indicating the number of significantly differentially expressed genes (DEGs) found in different detailed conditions from *in vivo* and *in vitro*.
b) Venn diagrams of the DEGs in the condition of compartment (oral and aboral) with other biology variables unfolded between *in vivo* and *in vitro* as in a.
c) Venn diagrams of the DEGs in the condition of gender (male and female) with other biology variables unfolded between *in vivo* and *in vitro* as in a.
d) Venn diagrams of the DEGs in the condition of aging (young and old) with other biology variables unfolded between *in vivo* and *in vitro* as in a.

Figure S5. Meta-analysis on specific biology features (compartment, gender, aging) respectively revealed how biology features changed in culture. Related to Figure3.
a, b) Hierarchical clustering and heatmap of the gene overlap correlation of pathways significantly enriched in compartment differences *in vivo* (a) and *in vitro* (b). Pathways sharing common genes could be clustered to super functional categories.

Figure S6. Meta-analysis on specific biology features (compartment, gender, aging) respectively revealed how biology features changed in culture. Related to Figure3.
a, b) Hierarchical clustering and heatmap of the gene overlap correlation of pathways significantly enriched in gender differences *in vivo* (a) and *in vitro* (b). Pathways sharing common genes could be clustered to super functional categories.

Figure S7. Meta-analysis on specific biology features (compartment, gender, aging) respectively revealed how biology features changed in culture. Related to Figure3.
a, b) Hierarchical clustering and heatmap of the gene overlap correlation of pathways significantly enriched in aging differences *in vivo* (a) and *in vitro* (b). Pathways sharing common genes could be clustered to super functional categories.

Figure S8. Meta-analysis on specific biology features (compartment, gender, aging) respectively revealed how biology features changed in culture. Related to Figure3.
Bubble plots of candidate upstream regulators inducing the changes of compartment, gender and aging *in vivo* (left panel) and *in vitro* (right panel).

Figure S9. Characterization of organoids as an *in vitro* model for aging research. Related to Figure4.
a) Treemap overview of all significantly enriched pathways in aging *in vivo*, which were kept in culture in at least one condition.
b) Box plot of the number of conditions of each aging-enriched pathway kept in culture with line connected between the pathway *in vivo* and that *in vitro* by using the same datasets as in a.
c) Treemap overview of all significantly enriched pathways in aging *in vivo*, which were lost in culture.

Supplementary Tables

Table S1. *In vitro* culturing of intestinal crypts as organoids does substantially affect their transcriptional program. Related to Figure1.
List of highly contributed genes to PC1-PC6 from PCA analysis (genes with log2(var_contrib) more than -3.3 were selected) and GO terms of PC-enriched biology processes (GO terms with p value less than 0.01 were selected).

Table S2. Cell population composition in all the conditions illustrated higher proportion of stem/TA cells *in vitro* than those *in vivo*. Related to Figure2.
Cell population composition analysis in crypts and organoids including DEGs between crypts and organoids, cell type markers used for GSEA, trained feature model for CIBERSORTx, cell type deconvolution results from simulated, real-world data and bulk RNA-seq data in this paper.

Table S3. Meta-analysis on specific biology features respectively revealed how biology features changed in culture. Related to Figure3.
Canonical pathway analysis of the differentially expressed genes in compartment, gender, aging with *in vivo* and *in vitro* separated.

Table S4. Detailed investigation for organoids as an *in vitro* model on aging demonstrated only a small proportion of aging pathways in some specific conditions could be studied *in vitro*. Related to Figure4.
Aging pathways kept or lost after culture in different conditions of compartment and gender. Pathways with adjusted p value less than 0.05 were selected.

Figure S1

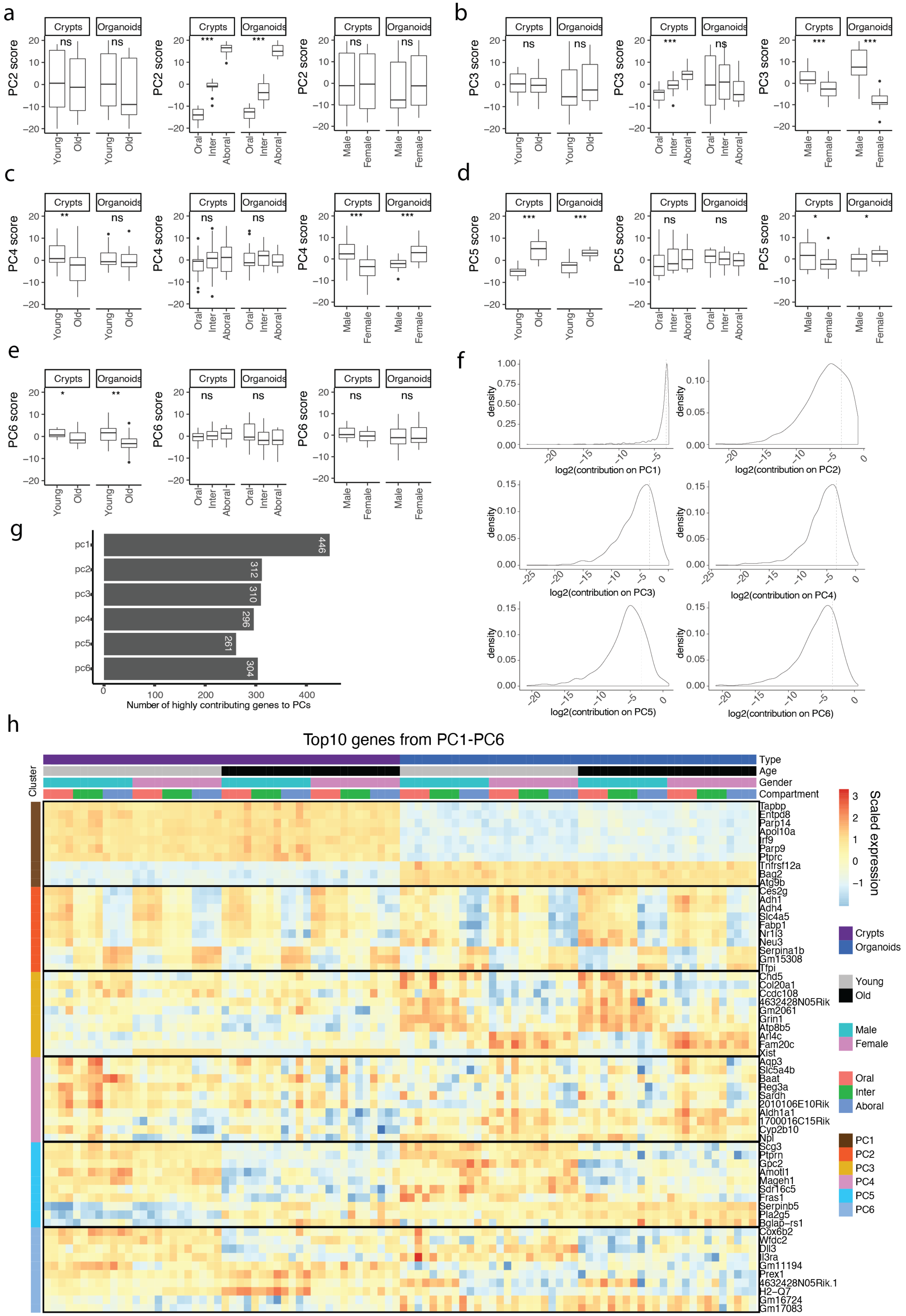

a
b
c
d
e
f
g
h
Crypts
Organoids
PC2 score
PC3 score
PC4 score
PC5 score
PC6 score
ns
*
**
***
Young
Old
Oral
Inter
Aboral
Male
Female
density
log2(contribution on PC1)
log2(contribution on PC2)
log2(contribution on PC3)
log2(contribution on PC4)
log2(contribution on PC5)
log2(contribution on PC6)
pc1
pc2
pc3
pc4
pc5
pc6
446
312
310
296
261
304
Number of highly contributing genes to PCs
Top10 genes from PC1-PC6
Cluster
Type
Age
Gender
Compartment
Tapbp
Entpd8
Parp14
Apol10a
Irf9
Parp9
Ptprc
Tnfrsf12a
Bag2
Atg9b
Ces2g
Adh1
Adh4
Slc4a5
Fabp1
Nr1i3
Neu3
Serpina1b
Gm15308
Tfpi
Chd5
Col20a1
Ccdc108
4632428N05Rik
Gm2061
Grin1
Atp8b5
Arl4c
Fam20c
Xist
Aqp3
Slc5a4b
Baat
Reg3a
Sardh
2010106E10Rik
Aldh1a1
1700016C15Rik
Cyp2b10
Npl
Scg3
Ptprn
Gpc2
Amotl1
Mageh1
Sdr16c5
Fras1
Serpinb5
Pla2g5
Bglap−rs1
Cox6b2
Wfdc2
Dll3
Il3ra
Gm11194
Prex1
4632428N05Rik.1
H2−Q7
Gm16724
Gm17083
Scaled expression
PC1
PC2
PC3
PC4
PC5
PC6

Figure S2

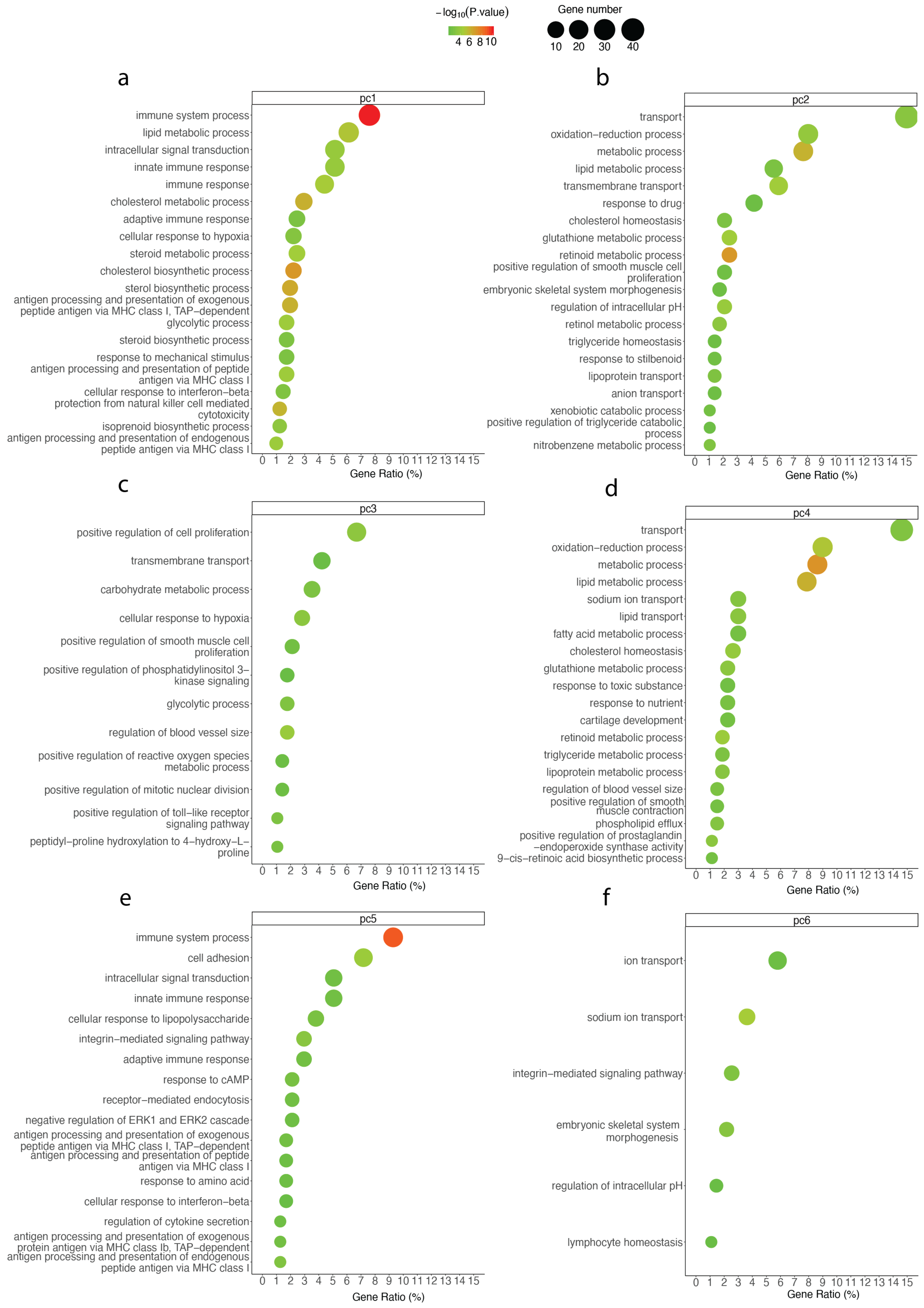
−log10(P.value)
4 6 8 10
Gene number
10 20 30 40
a
pc1
immune system process
lipid metabolic process
intracellular signal transduction
innate immune response
immune response
cholesterol metabolic process
adaptive immune response
cellular response to hypoxia
steroid metabolic process
cholesterol biosynthetic process
sterol biosynthetic process
antigen processing and presentation of exogenous peptide antigen via MHC class I, TAP−dependent
glycolytic process
steroid biosynthetic process
response to mechanical stimulus
antigen processing and presentation of peptide antigen via MHC class I
cellular response to interferon−beta
protection from natural killer cell mediated cytotoxicity
isoprenoid biosynthetic process
antigen processing and presentation of endogenous peptide antigen via MHC class I
0 1 2 3 4 5 6 7 8 9 10 11 12 13 14 15
Gene Ratio (%)
b
pc2
transport
oxidation−reduction process
metabolic process
lipid metabolic process
transmembrane transport
response to drug
cholesterol homeostasis
glutathione metabolic process
retinoid metabolic process
positive regulation of smooth muscle cell proliferation
embryonic skeletal system morphogenesis
regulation of intracellular pH
retinol metabolic process
triglyceride homeostasis
response to stilbenoid
lipoprotein transport
anion transport
xenobiotic catabolic process
positive regulation of triglyceride catabolic process
nitrobenzene metabolic process
0 1 2 3 4 5 6 7 8 9 10 11 12 13 14 15
Gene Ratio (%)
c
pc3
positive regulation of cell proliferation
transmembrane transport
carbohydrate metabolic process
cellular response to hypoxia
positive regulation of smooth muscle cell proliferation
positive regulation of phosphatidylinositol 3−kinase signaling
glycolytic process
regulation of blood vessel size
positive regulation of reactive oxygen species metabolic process
positive regulation of mitotic nuclear division
positive regulation of toll−like receptor signaling pathway
peptidyl−proline hydroxylation to 4−hydroxy−L−proline
0 1 2 3 4 5 6 7 8 9 10 11 12 13 14 15
Gene Ratio (%)
d
pc4
transport
oxidation−reduction process
metabolic process
lipid metabolic process
sodium ion transport
lipid transport
fatty acid metabolic process
cholesterol homeostasis
glutathione metabolic process
response to toxic substance
response to nutrient
cartilage development
retinoid metabolic process
triglyceride metabolic process
lipoprotein metabolic process
regulation of blood vessel size
positive regulation of smooth muscle contraction
phospholipid efflux
positive regulation of prostaglandin−endoperoxide synthase activity
9−cis−retinoic acid biosynthetic process
0 1 2 3 4 5 6 7 8 9 10 11 12 13 14 15
Gene Ratio (%)
e
pc5
immune system process
cell adhesion
intracellular signal transduction
innate immune response
cellular response to lipopolysaccharide
integrin−mediated signaling pathway
adaptive immune response
response to cAMP
receptor−mediated endocytosis
negative regulation of ERK1 and ERK2 cascade
antigen processing and presentation of exogenous peptide antigen via MHC class I, TAP−dependent
antigen processing and presentation of peptide antigen via MHC class I
response to amino acid
cellular response to interferon−beta
regulation of cytokine secretion
antigen processing and presentation of exogenous protein antigen via MHC class Ib, TAP−dependent
antigen processing and presentation of endogenous peptide antigen via MHC class I
0 1 2 3 4 5 6 7 8 9 10 11 12 13 14 15
Gene Ratio (%)
f
pc6
ion transport
sodium ion transport
integrin−mediated signaling pathway
embryonic skeletal system morphogenesis
regulation of intracellular pH
lymphocyte homeostasis
0 1 2 3 4 5 6 7 8 9 10 11 12 13 14 15
Gene Ratio (%)

Figure S3

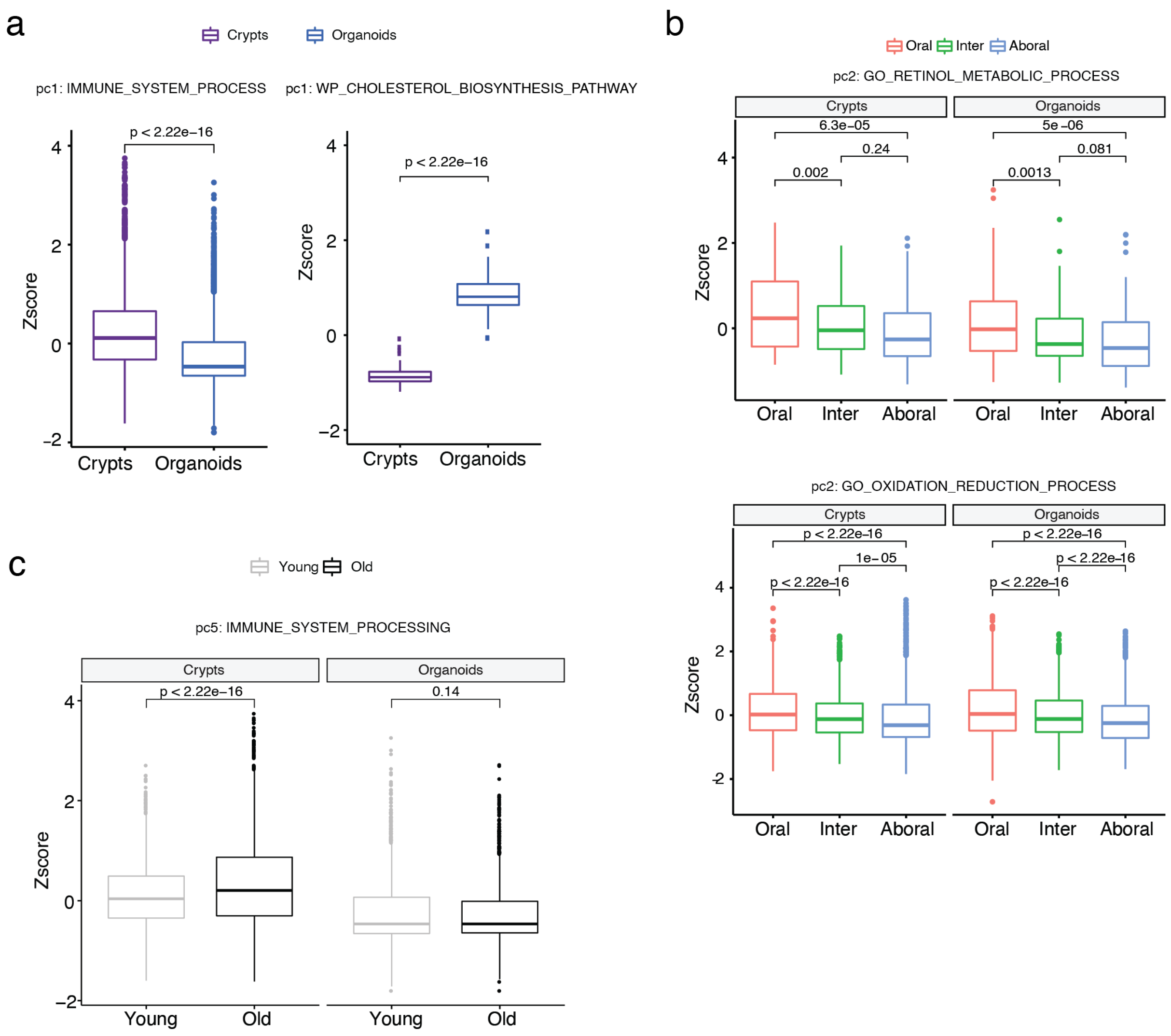
a
Crypts
Organoids
pc1: IMMUNE_SYSTEM_PROCESS
pc1: WP_CHOLESTEROL_BIOSYNTHESIS_PATHWAY
p < 2.22e−16
p < 2.22e−16
Zscore
Crypts
Organoids
b
Oral
Inter
Aboral
pc2: GO_RETINOL_METABOLIC_PROCESS
Crypts
Organoids
6.3e−05
0.24
0.002
5e−06
0.081
0.0013
Zscore
pc2: GO_OXIDATION_REDUCTION_PROCESS
p < 2.22e−16
1e−05
p < 2.22e−16
c
Young
Old
pc5: IMMUNE_SYSTEM_PROCESSING
Crypts
Organoids
p < 2.22e−16
0.14
Zscore
Young
Old

Figure S4

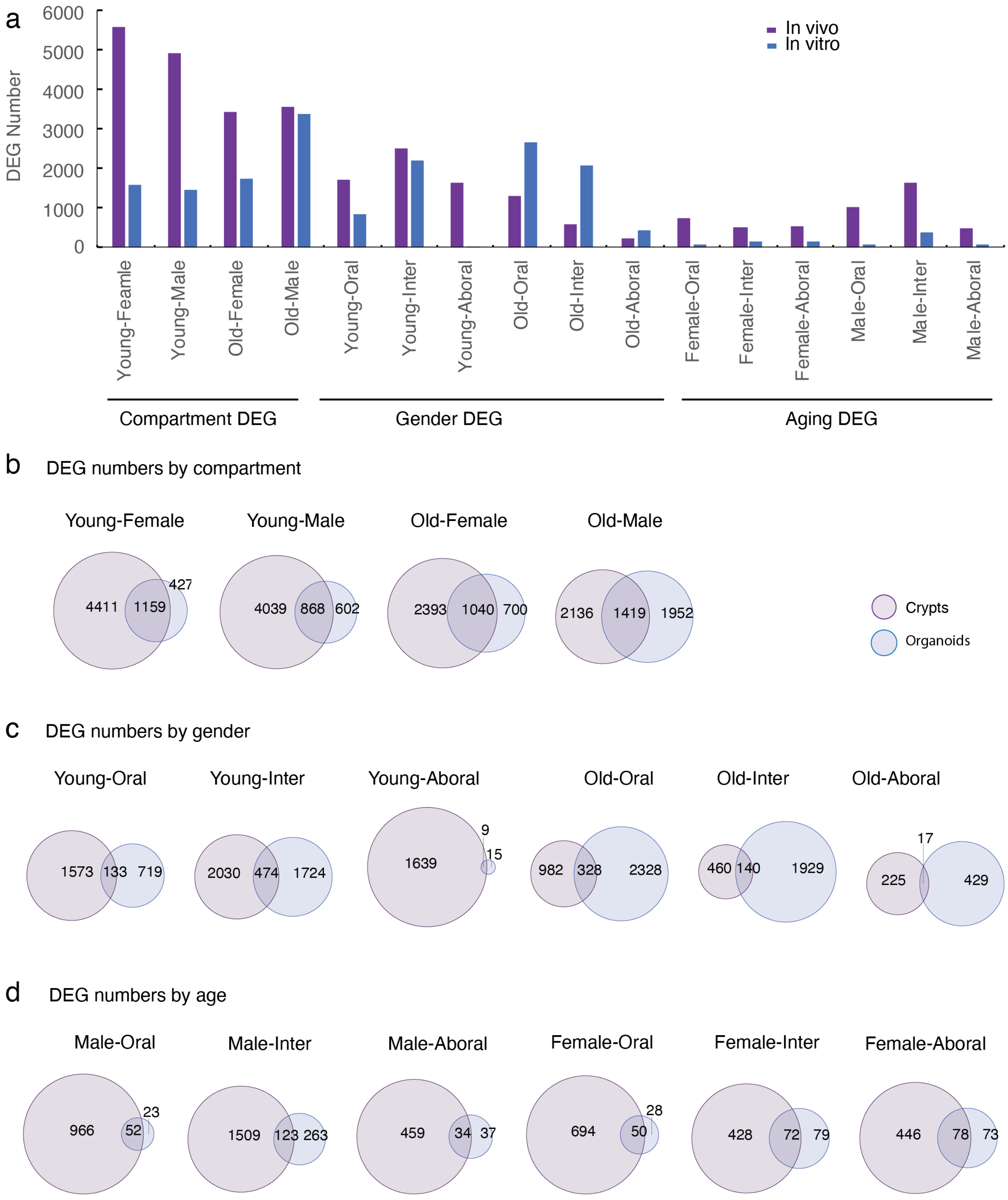
a
DEG Number
6000
5000
4000
3000
2000
1000
0
In vivo
In vitro
Young-Feamle
Young-Male
Old-Female
Old-Male
Young-Oral
Young-Inter
Young-Aboral
Old-Oral
Old-Inter
Old-Aboral
Female-Oral
Female-Inter
Female-Aboral
Male-Oral
Male-Inter
Male-Aboral
Compartment DEG
Gender DEG
Aging DEG
b
DEG numbers by compartment
Young-Female
4411
1159
427
Young-Male
4039
868
602
Old-Female
2393
1040
700
Old-Male
2136
1419
1952
Crypts
Organoids
c
DEG numbers by gender
Young-Oral
1573
133
719
Young-Inter
2030
474
1724
Young-Aboral
1639
9
15
Old-Oral
982
328
2328
Old-Inter
460
140
1929
Old-Aboral
225
17
429
d
DEG numbers by age
Male-Oral
966
52
23
Male-Inter
1509
123
263
Male-Aboral
459
34
37
Female-Oral
694
50
28
Female-Inter
428
72
79
Female-Aboral
446
78
73

Figure S5

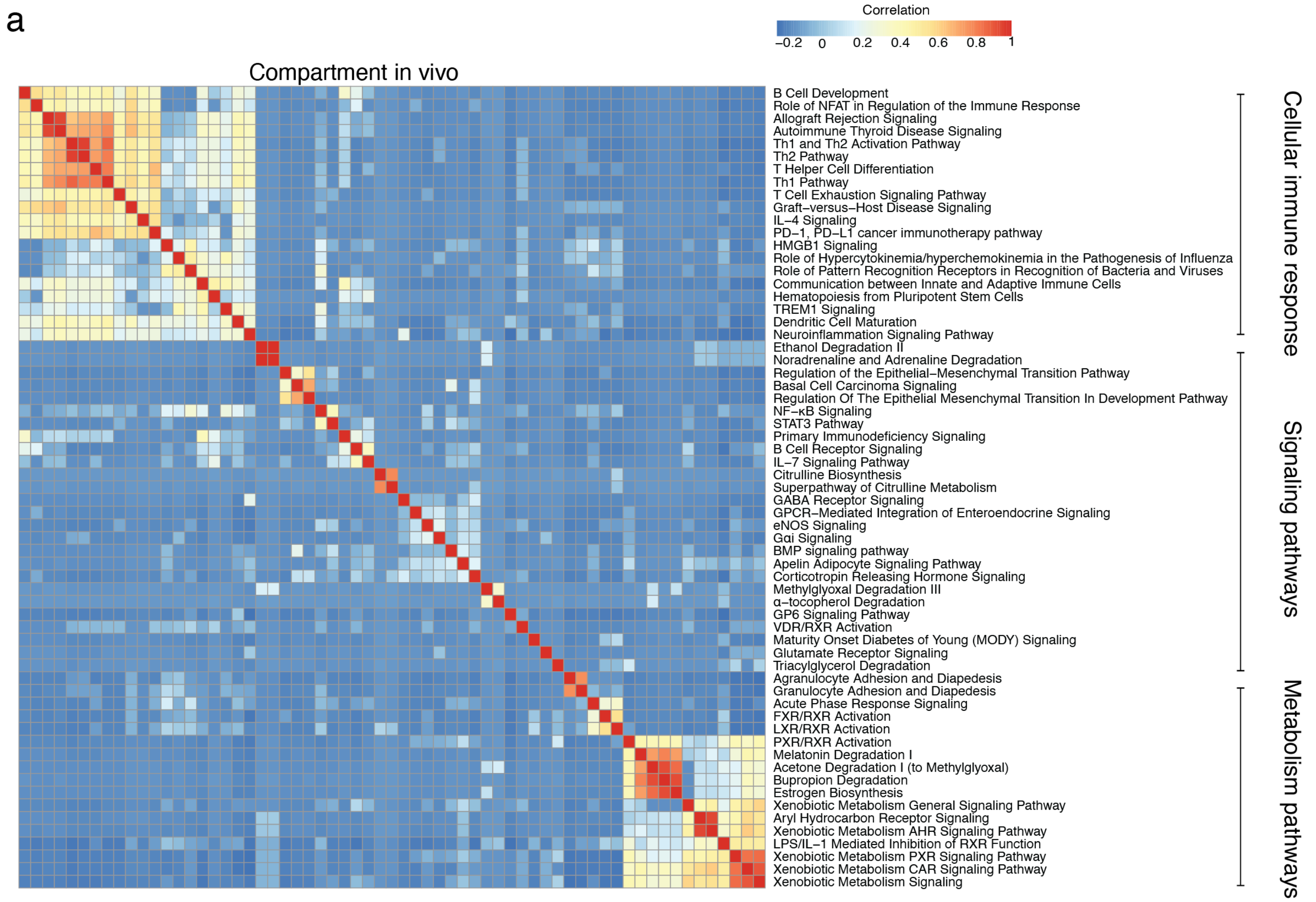
a
Correlation
−0.2
0
0.2
0.4
0.6
0.8
1
Compartment in vivo
B Cell Development
Role of NFAT in Regulation of the Immune Response
Allograft Rejection Signaling
Autoimmune Thyroid Disease Signaling
Th1 and Th2 Activation Pathway
Th2 Pathway
T Helper Cell Differentiation
Th1 Pathway
T Cell Exhaustion Signaling Pathway
Graft−versus−Host Disease Signaling
IL−4 Signaling
PD−1, PD−L1 cancer immunotherapy pathway
HMGB1 Signaling
Role of Hypercytokinemia/hyperchemokinemia in the Pathogenesis of Influenza
Role of Pattern Recognition Receptors in Recognition of Bacteria and Viruses
Communication between Innate and Adaptive Immune Cells
Hematopoiesis from Pluripotent Stem Cells
TREM1 Signaling
Dendritic Cell Maturation
Neuroinflammation Signaling Pathway
Ethanol Degradation II
Noradrenaline and Adrenaline Degradation
Regulation of the Epithelial−Mesenchymal Transition Pathway
Basal Cell Carcinoma Signaling
Regulation Of The Epithelial Mesenchymal Transition In Development Pathway
NF−κB Signaling
STAT3 Pathway
Primary Immunodeficiency Signaling
B Cell Receptor Signaling
IL−7 Signaling Pathway
Citrulline Biosynthesis
Superpathway of Citrulline Metabolism
GABA Receptor Signaling
GPCR−Mediated Integration of Enteroendocrine Signaling
eNOS Signaling
Gαi Signaling
BMP signaling pathway
Apelin Adipocyte Signaling Pathway
Corticotropin Releasing Hormone Signaling
Methylglyoxal Degradation III
α−tocopherol Degradation
GP6 Signaling Pathway
VDR/RXR Activation
Maturity Onset Diabetes of Young (MODY) Signaling
Glutamate Receptor Signaling
Triacylglycerol Degradation
Agranulocyte Adhesion and Diapedesis
Granulocyte Adhesion and Diapedesis
Acute Phase Response Signaling
FXR/RXR Activation
LXR/RXR Activation
PXR/RXR Activation
Melatonin Degradation I
Acetone Degradation I (to Methylglyoxal)
Bupropion Degradation
Estrogen Biosynthesis
Xenobiotic Metabolism General Signaling Pathway
Aryl Hydrocarbon Receptor Signaling
Xenobiotic Metabolism AHR Signaling Pathway
LPS/IL−1 Mediated Inhibition of RXR Function
Xenobiotic Metabolism PXR Signaling Pathway
Xenobiotic Metabolism CAR Signaling Pathway
Xenobiotic Metabolism Signaling
Cellular immune response
Signaling pathways
Metabolism pathways

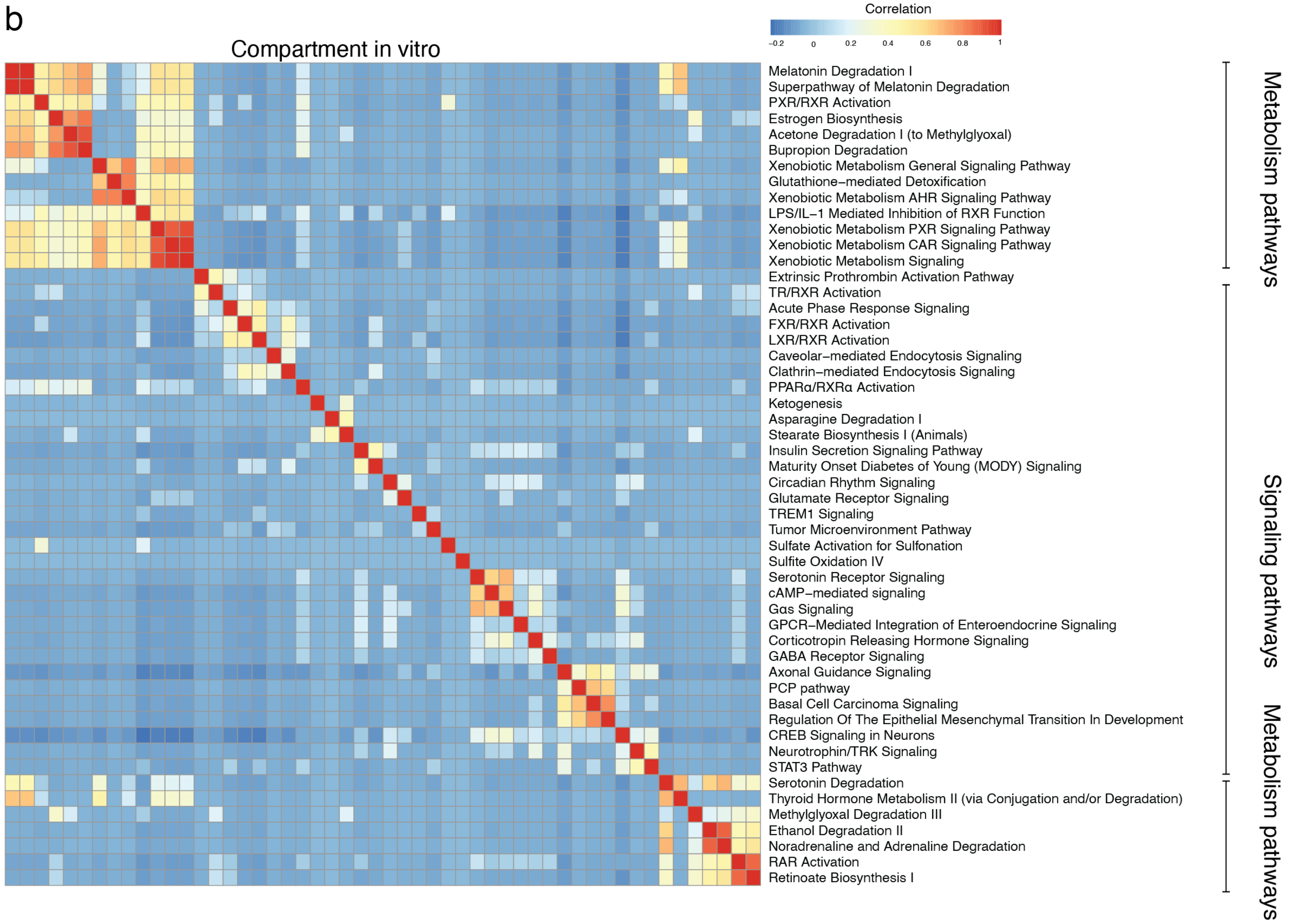
b
Correlation
−0.2
0
0.2
0.4
0.6
0.8
1
Compartment in vitro
Melatonin Degradation I
Superpathway of Melatonin Degradation
PXR/RXR Activation
Estrogen Biosynthesis
Acetone Degradation I (to Methylglyoxal)
Bupropion Degradation
Xenobiotic Metabolism General Signaling Pathway
Glutathione−mediated Detoxification
Xenobiotic Metabolism AHR Signaling Pathway
LPS/IL−1 Mediated Inhibition of RXR Function
Xenobiotic Metabolism PXR Signaling Pathway
Xenobiotic Metabolism CAR Signaling Pathway
Xenobiotic Metabolism Signaling
Extrinsic Prothrombin Activation Pathway
TR/RXR Activation
Acute Phase Response Signaling
FXR/RXR Activation
LXR/RXR Activation
Caveolar−mediated Endocytosis Signaling
Clathrin−mediated Endocytosis Signaling
PPARα/RXRα Activation
Ketogenesis
Asparagine Degradation I
Stearate Biosynthesis I (Animals)
Insulin Secretion Signaling Pathway
Maturity Onset Diabetes of Young (MODY) Signaling
Circadian Rhythm Signaling
Glutamate Receptor Signaling
TREM1 Signaling
Tumor Microenvironment Pathway
Sulfate Activation for Sulfonation
Sulfite Oxidation IV
Serotonin Receptor Signaling
cAMP−mediated signaling
Gαs Signaling
GPCR−Mediated Integration of Enteroendocrine Signaling
Corticotropin Releasing Hormone Signaling
GABA Receptor Signaling
Axonal Guidance Signaling
PCP pathway
Basal Cell Carcinoma Signaling
Regulation Of The Epithelial Mesenchymal Transition In Development
CREB Signaling in Neurons
Neurotrophin/TRK Signaling
STAT3 Pathway
Serotonin Degradation
Thyroid Hormone Metabolism II (via Conjugation and/or Degradation)
Methylglyoxal Degradation III
Ethanol Degradation II
Noradrenaline and Adrenaline Degradation
RAR Activation
Retinoate Biosynthesis I
Metabolism pathways
Signaling pathways
Metabolism pathways

Figure S6

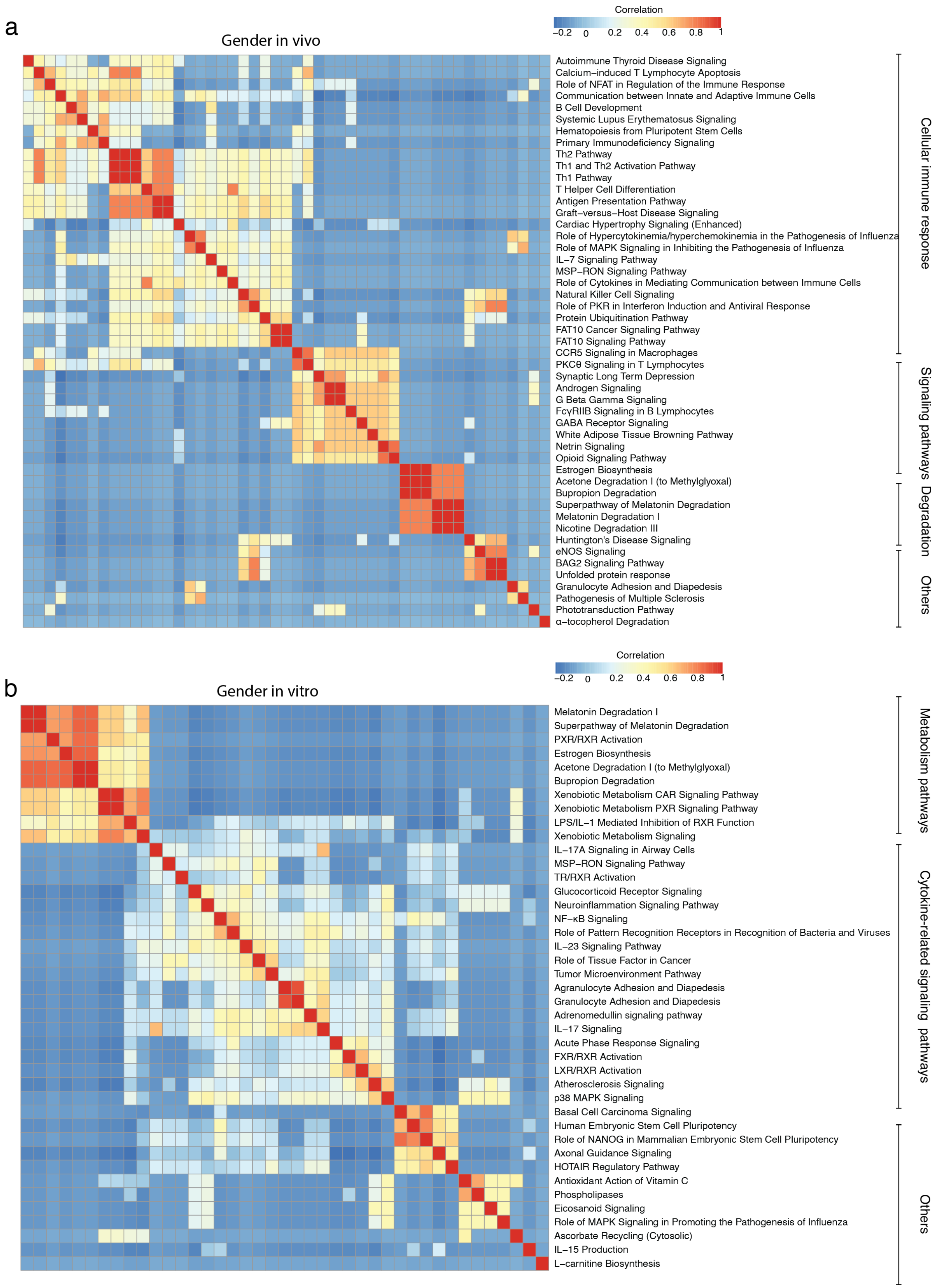
a
Gender in vivo
Correlation
−0.2
0
0.2
0.4
0.6
0.8
1
Autoimmune Thyroid Disease Signaling
Calcium−induced T Lymphocyte Apoptosis
Role of NFAT in Regulation of the Immune Response
Communication between Innate and Adaptive Immune Cells
B Cell Development
Systemic Lupus Erythematosus Signaling
Hematopoiesis from Pluripotent Stem Cells
Primary Immunodeficiency Signaling
Th2 Pathway
Th1 and Th2 Activation Pathway
Th1 Pathway
T Helper Cell Differentiation
Antigen Presentation Pathway
Graft−versus−Host Disease Signaling
Cardiac Hypertrophy Signaling (Enhanced)
Role of Hypercytokinemia/hyperchemokinemia in the Pathogenesis of Influenza
Role of MAPK Signaling in Inhibiting the Pathogenesis of Influenza
IL−7 Signaling Pathway
MSP−RON Signaling Pathway
Role of Cytokines in Mediating Communication between Immune Cells
Natural Killer Cell Signaling
Role of PKR in Interferon Induction and Antiviral Response
Protein Ubiquitination Pathway
FAT10 Cancer Signaling Pathway
FAT10 Signaling Pathway
CCR5 Signaling in Macrophages
PKCθ Signaling in T Lymphocytes
Synaptic Long Term Depression
Androgen Signaling
G Beta Gamma Signaling
FcγRIIB Signaling in B Lymphocytes
GABA Receptor Signaling
White Adipose Tissue Browning Pathway
Netrin Signaling
Opioid Signaling Pathway
Estrogen Biosynthesis
Acetone Degradation I (to Methylglyoxal)
Bupropion Degradation
Superpathway of Melatonin Degradation
Melatonin Degradation I
Nicotine Degradation III
Huntington's Disease Signaling
eNOS Signaling
BAG2 Signaling Pathway
Unfolded protein response
Granulocyte Adhesion and Diapedesis
Pathogenesis of Multiple Sclerosis
Phototransduction Pathway
α−tocopherol Degradation
Cellular immune response
Signaling pathways
Degradation
Others
b
Gender in vitro
Correlation
−0.2
0
0.2
0.4
0.6
0.8
1
Melatonin Degradation I
Superpathway of Melatonin Degradation
PXR/RXR Activation
Estrogen Biosynthesis
Acetone Degradation I (to Methylglyoxal)
Bupropion Degradation
Xenobiotic Metabolism CAR Signaling Pathway
Xenobiotic Metabolism PXR Signaling Pathway
LPS/IL−1 Mediated Inhibition of RXR Function
Xenobiotic Metabolism Signaling
IL−17A Signaling in Airway Cells
MSP−RON Signaling Pathway
TR/RXR Activation
Glucocorticoid Receptor Signaling
Neuroinflammation Signaling Pathway
NF−κB Signaling
Role of Pattern Recognition Receptors in Recognition of Bacteria and Viruses
IL−23 Signaling Pathway
Role of Tissue Factor in Cancer
Tumor Microenvironment Pathway
Agranulocyte Adhesion and Diapedesis
Granulocyte Adhesion and Diapedesis
Adrenomedullin signaling pathway
IL−17 Signaling
Acute Phase Response Signaling
FXR/RXR Activation
LXR/RXR Activation
Atherosclerosis Signaling
p38 MAPK Signaling
Basal Cell Carcinoma Signaling
Human Embryonic Stem Cell Pluripotency
Role of NANOG in Mammalian Embryonic Stem Cell Pluripotency
Axonal Guidance Signaling
HOTAIR Regulatory Pathway
Antioxidant Action of Vitamin C
Phospholipases
Eicosanoid Signaling
Role of MAPK Signaling in Promoting the Pathogenesis of Influenza
Ascorbate Recycling (Cytosolic)
IL−15 Production
L−carnitine Biosynthesis
Metabolism pathways
Cytokine-related signaling pathways
Others

Figure S7

a

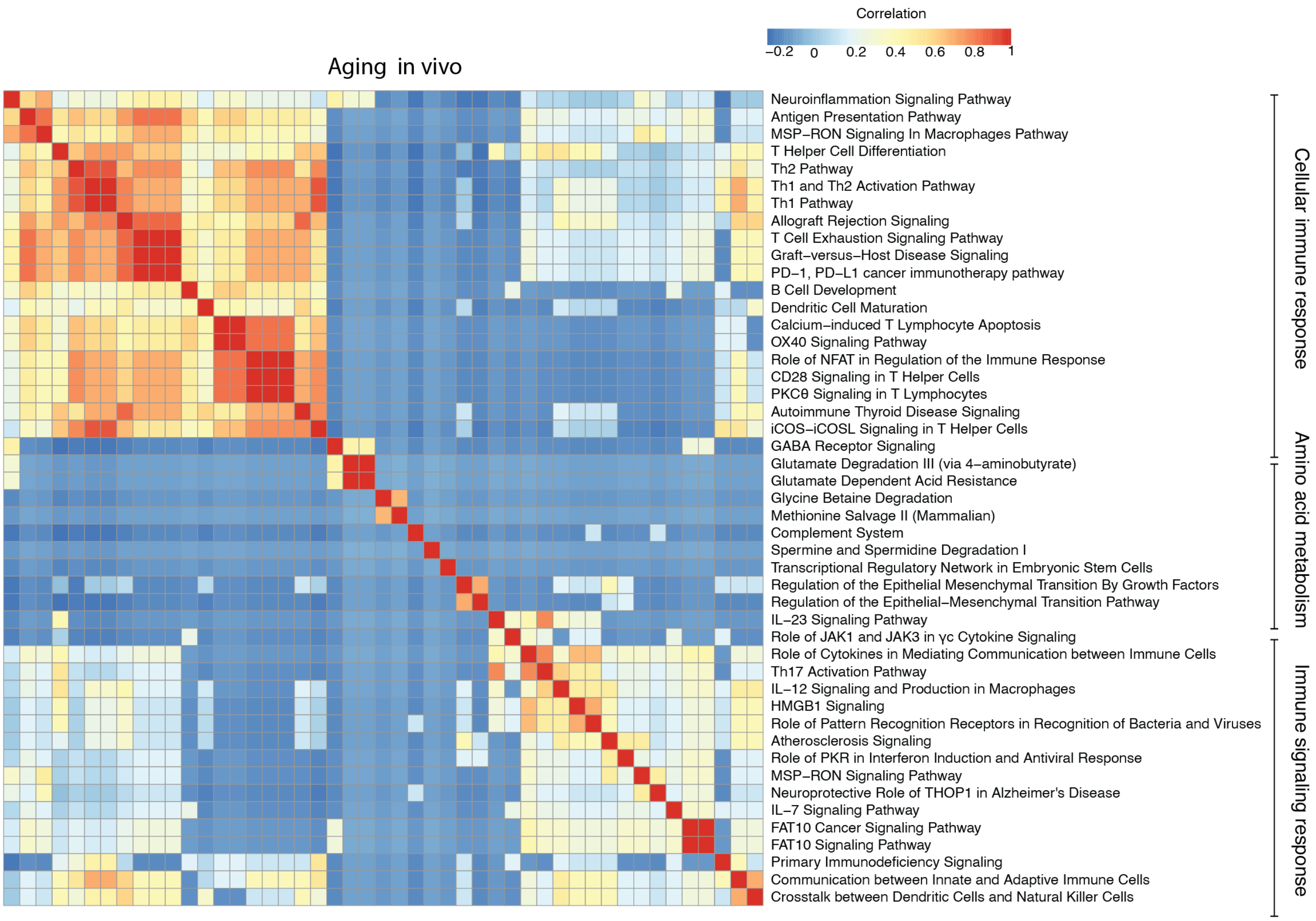

Aging in vivo
Correlation
−0.2 0 0.2 0.4 0.6 0.8 1
Neuroinflammation Signaling Pathway
Antigen Presentation Pathway
MSP−RON Signaling In Macrophages Pathway
T Helper Cell Differentiation
Th2 Pathway
Th1 and Th2 Activation Pathway
Th1 Pathway
Allograft Rejection Signaling
T Cell Exhaustion Signaling Pathway
Graft−versus−Host Disease Signaling
PD−1, PD−L1 cancer immunotherapy pathway
B Cell Development
Dendritic Cell Maturation
Calcium−induced T Lymphocyte Apoptosis
OX40 Signaling Pathway
Role of NFAT in Regulation of the Immune Response
CD28 Signaling in T Helper Cells
PKCθ Signaling in T Lymphocytes
Autoimmune Thyroid Disease Signaling
iCOS−iCOSL Signaling in T Helper Cells
GABA Receptor Signaling
Glutamate Degradation III (via 4−aminobutyrate)
Glutamate Dependent Acid Resistance
Glycine Betaine Degradation
Methionine Salvage II (Mammalian)
Complement System
Spermine and Spermidine Degradation I
Transcriptional Regulatory Network in Embryonic Stem Cells
Regulation of the Epithelial Mesenchymal Transition By Growth Factors
Regulation of the Epithelial−Mesenchymal Transition Pathway
IL−23 Signaling Pathway
Role of JAK1 and JAK3 in γc Cytokine Signaling
Role of Cytokines in Mediating Communication between Immune Cells
Th17 Activation Pathway
IL−12 Signaling and Production in Macrophages
HMGB1 Signaling
Role of Pattern Recognition Receptors in Recognition of Bacteria and Viruses
Atherosclerosis Signaling
Role of PKR in Interferon Induction and Antiviral Response
MSP−RON Signaling Pathway
Neuroprotective Role of THOP1 in Alzheimer's Disease
IL−7 Signaling Pathway
FAT10 Cancer Signaling Pathway
FAT10 Signaling Pathway
Primary Immunodeficiency Signaling
Communication between Innate and Adaptive Immune Cells
Crosstalk between Dendritic Cells and Natural Killer Cells
Cellular immune response
Amino acid metabolism
Immune signaling response

b

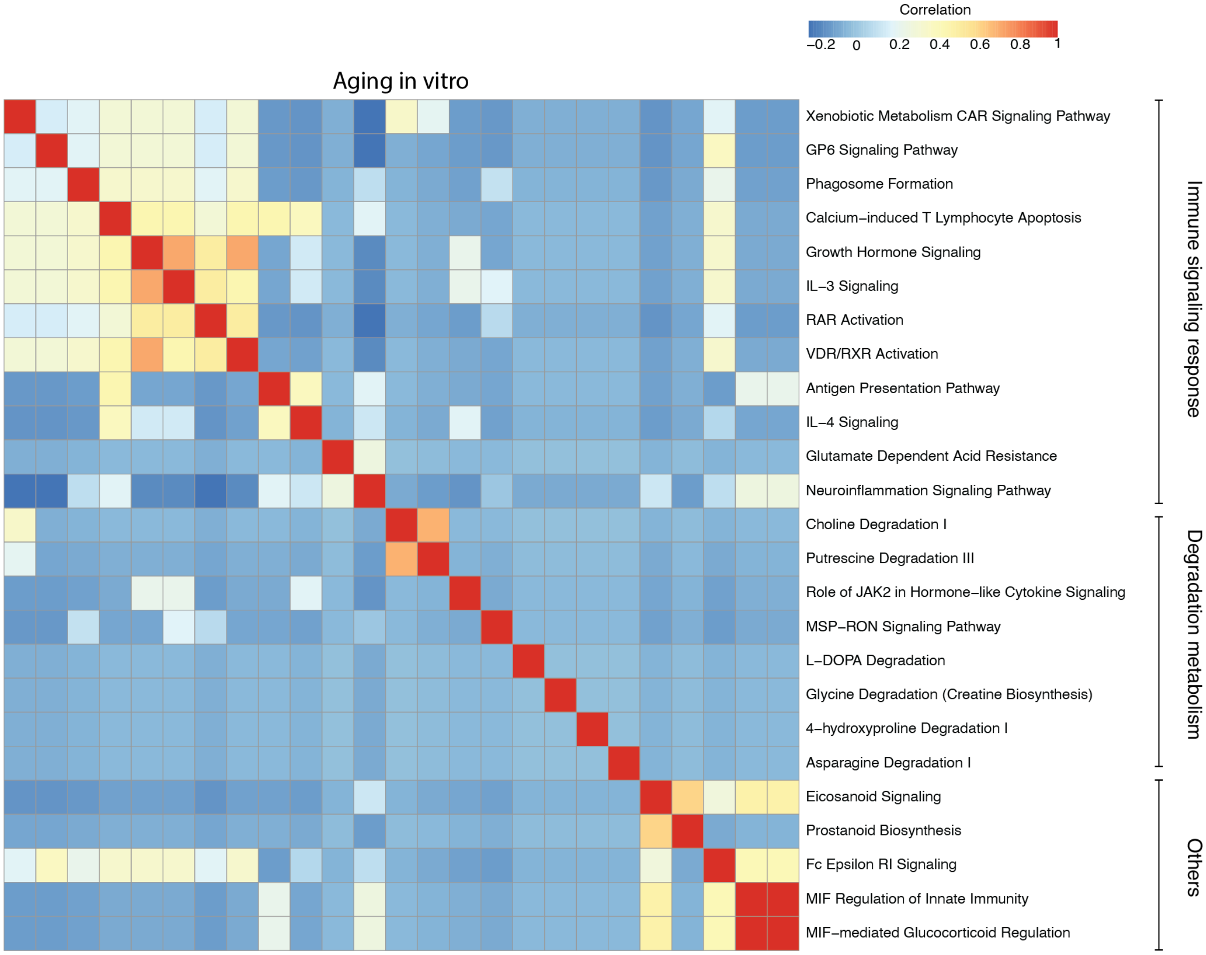

Aging in vitro
Correlation
−0.2 0 0.2 0.4 0.6 0.8 1
Xenobiotic Metabolism CAR Signaling Pathway
GP6 Signaling Pathway
Phagosome Formation
Calcium−induced T Lymphocyte Apoptosis
Growth Hormone Signaling
IL−3 Signaling
RAR Activation
VDR/RXR Activation
Antigen Presentation Pathway
IL−4 Signaling
Glutamate Dependent Acid Resistance
Neuroinflammation Signaling Pathway
Choline Degradation I
Putrescine Degradation III
Role of JAK2 in Hormone−like Cytokine Signaling
MSP−RON Signaling Pathway
L−DOPA Degradation
Glycine Degradation (Creatine Biosynthesis)
4−hydroxyproline Degradation I
Asparagine Degradation I
Eicosanoid Signaling
Prostanoid Biosynthesis
Fc Epsilon RI Signaling
MIF Regulation of Innate Immunity
MIF−mediated Glucocorticoid Regulation
Immune signaling response
Degradation metabolism
Others

Figure S8

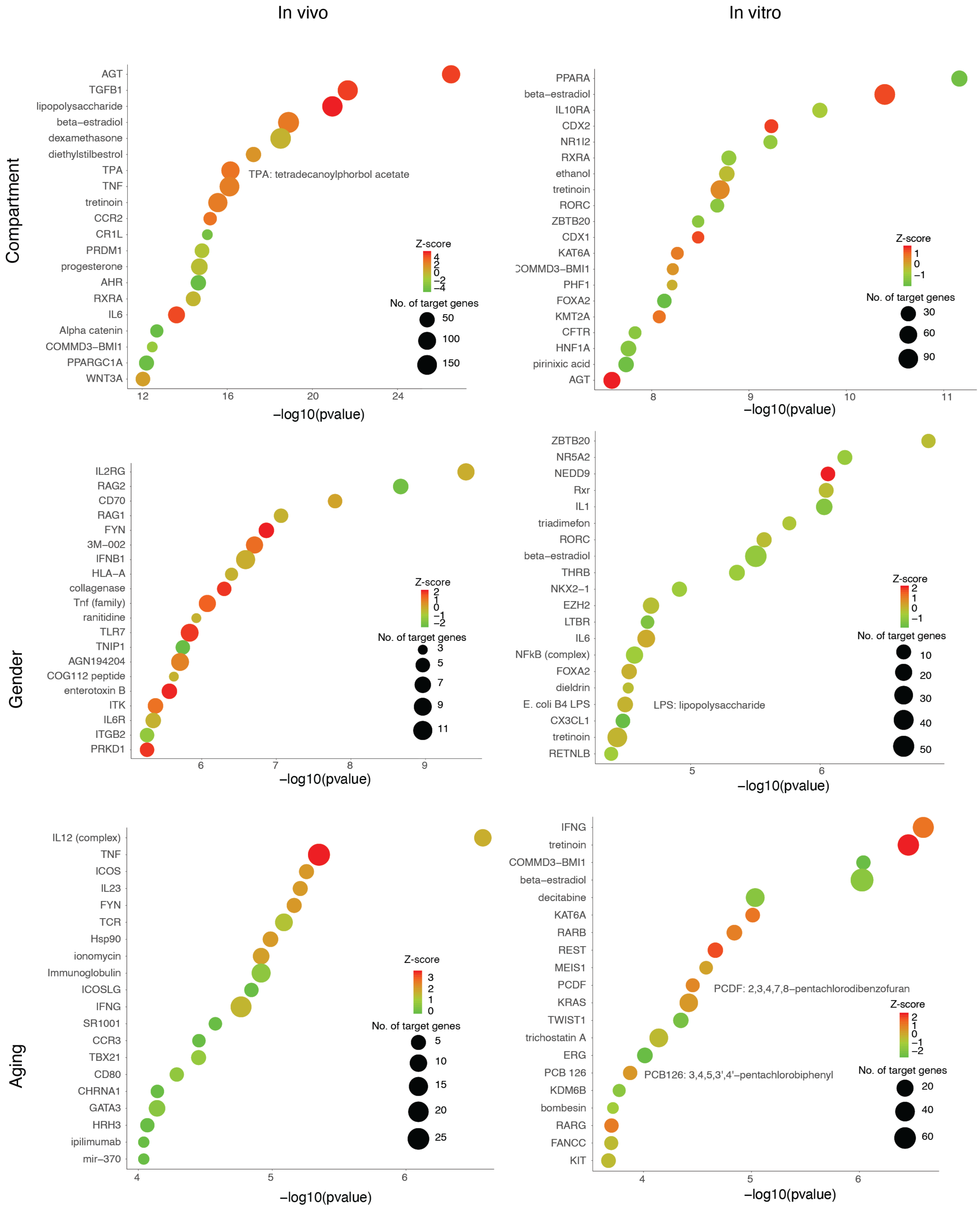
In vivo
In vitro
Compartment
Gender
Aging
AGT
TGFB1
lipopolysaccharide
beta−estradiol
dexamethasone
diethylstilbestrol
TPA
TNF
tretinoin
CCR2
CR1L
PRDM1
progesterone
AHR
RXRA
IL6
Alpha catenin
COMMD3−BMI1
PPARGC1A
WNT3A
TPA: tetradecanoylphorbol acetate
Z-score
No. of target genes
−log10(pvalue)
PPARA
beta−estradiol
IL10RA
CDX2
NR1I2
RXRA
ethanol
tretinoin
RORC
ZBTB20
CDX1
KAT6A
COMMD3−BMI1
PHF1
FOXA2
KMT2A
CFTR
HNF1A
pirinixic acid
AGT
IL2RG
RAG2
CD70
RAG1
FYN
3M−002
IFNB1
HLA−A
collagenase
Tnf (family)
ranitidine
TLR7
TNIP1
AGN194204
COG112 peptide
enterotoxin B
ITK
IL6R
ITGB2
PRKD1
ZBTB20
NR5A2
NEDD9
Rxr
IL1
triadimefon
RORC
beta−estradiol
THRB
NKX2−1
EZH2
LTBR
IL6
NFkB (complex)
FOXA2
dieldrin
E. coli B4 LPS
CX3CL1
tretinoin
RETNLB
LPS: lipopolysaccharide
IL12 (complex)
TNF
ICOS
IL23
FYN
TCR
Hsp90
ionomycin
Immunoglobulin
ICOSLG
IFNG
SR1001
CCR3
TBX21
CD80
CHRNA1
GATA3
HRH3
ipilimumab
mir−370
IFNG
tretinoin
COMMD3−BMI1
beta−estradiol
decitabine
KAT6A
RARB
REST
MEIS1
PCDF
KRAS
TWIST1
trichostatin A
ERG
PCB 126
KDM6B
bombesin
RARG
FANCC
KIT
PCDF: 2,3,4,7,8−pentachlorodibenzofuran
PCB126: 3,4,5,3',4'−pentachlorobiphenyl

Figure S9

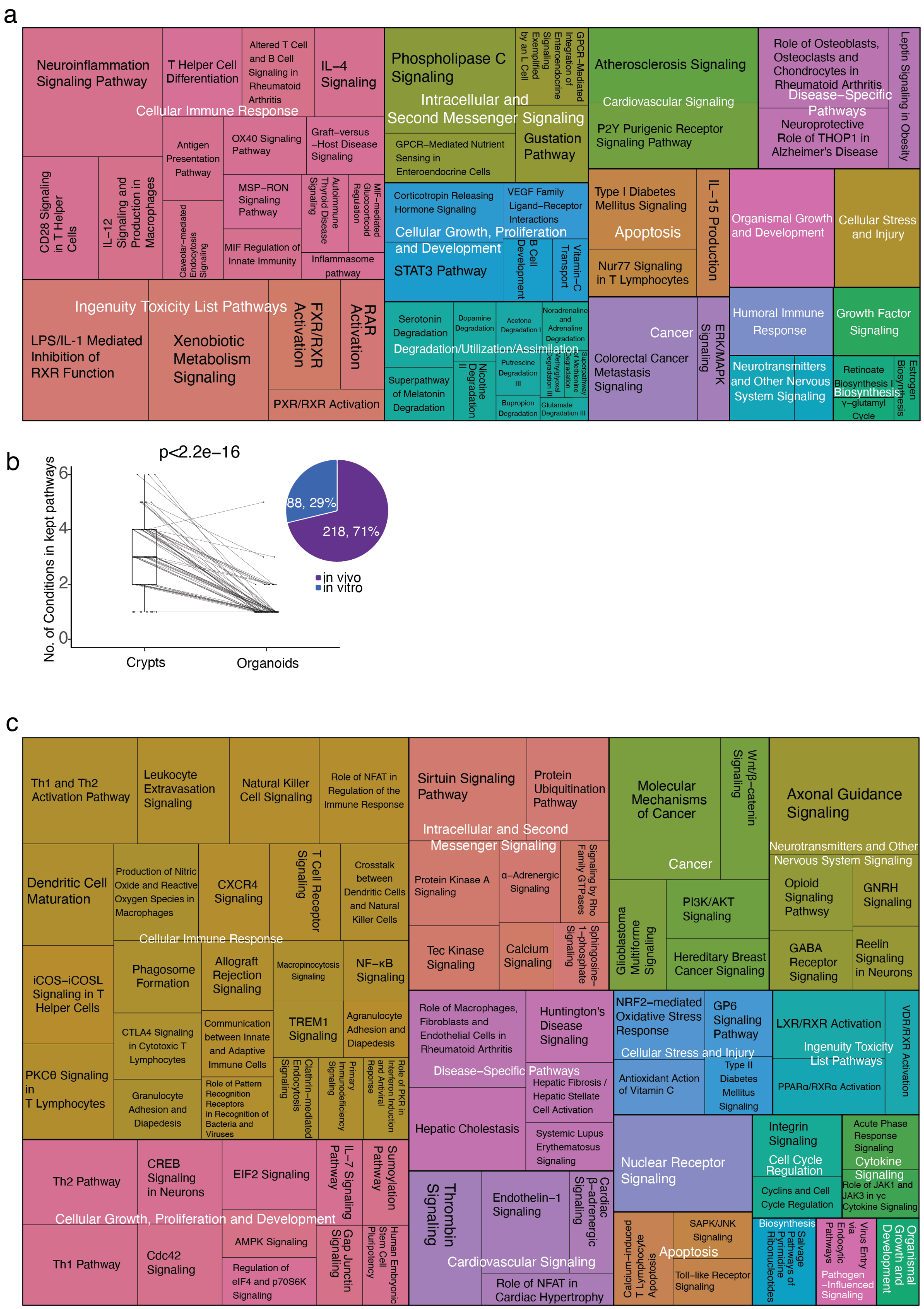
a
Neuroinflammation Signaling Pathway
T Helper Cell Differentiation
Altered T Cell and B Cell Signaling in Rheumatoid Arthritis
IL−4 Signaling
Cellular Immune Response
Phospholipase C Signaling
Intracellular and Second Messenger Signaling
GPCR−Mediated Integration of Enteroendocrine Signaling Exemplified by an L Cell
Atherosclerosis Signaling
Cardiovascular Signaling
P2Y Purigenic Receptor Signaling Pathway
Role of Osteoblasts, Osteoclasts and Chondrocytes in Rheumatoid Arthritis
Disease−Specific Pathways
Neuroprotective Role of THOP1 in Alzheimer's Disease
Leptin Signaling in Obesity
CD28 Signaling in T Helper Cells
IL−12 Signaling and Production in Macrophages
Antigen Presentation Pathway
OX40 Signaling Pathway
Graft−versus −Host Disease Signaling
GPCR−Mediated Nutrient Sensing in Enteroendocrine Cells
Gustation Pathway
Caveolar−mediated Endocytosis Signaling
MSP−RON Signaling Pathway
Autoimmune Thyroid Disease Signaling
MIF−mediated Glucocorticoid Regulation
MIF Regulation of Innate Immunity
Inflammasome pathway
Corticotropin Releasing Hormone Signaling
VEGF Family Ligand−Receptor Interactions
Cellular Growth, Proliferation and Development
STAT3 Pathway
B Cell Development
Vitamin−C Transport
Type I Diabetes Mellitus Signaling
Apoptosis
Nur77 Signaling in T Lymphocytes
IL−15 Production
Organismal Growth and Development
Cellular Stress and Injury
Ingenuity Toxicity List Pathways
LPS/IL−1 Mediated Inhibition of RXR Function
Xenobiotic Metabolism Signaling
FXR/RXR Activation
RAR Activation
PXR/RXR Activation
Serotonin Degradation
Dopamine Degradation
Acetone Degradation I
Noradrenaline and Adrenaline Degradation
Degradation/Utilization/Assimilation
Superpathway of Melatonin Degradation
Nicotine Degradation II
Putrescine Degradation III
Methylglyoxal Degradation III
Superpathway of Methionine Degradation
Bupropion Degradation
Glutamate Degradation III
Cancer
Colorectal Cancer Metastasis Signaling
ERK/MAPK Signaling
Humoral Immune Response
Growth Factor Signaling
Neurotransmitters and Other Nervous System Signaling
Retinoate Biosynthesis I
Biosynthesis
γ−glutamyl Cycle
Estrogen Biosynthesis
b
p<2.2e−16
No. of Conditions in kept pathways
0
2
4
6
Crypts
Organoids
88, 29%
218, 71%
in vivo
in vitro
c
Th1 and Th2 Activation Pathway
Leukocyte Extravasation Signaling
Natural Killer Cell Signaling
Role of NFAT in Regulation of the Immune Response
Sirtuin Signaling Pathway
Protein Ubiquitination Pathway
Intracellular and Second Messenger Signaling
Molecular Mechanisms of Cancer
Wnt/β−catenin Signaling
Cancer
Axonal Guidance Signaling
Neurotransmitters and Other Nervous System Signaling
Dendritic Cell Maturation
Production of Nitric Oxide and Reactive Oxygen Species in Macrophages
CXCR4 Signaling
T Cell Receptor Signaling
Crosstalk between Dendritic Cells and Natural Killer Cells
Protein Kinase A Signaling
α−Adrenergic Signaling
Signaling by Rho Family GTPases
Glioblastoma Multiforme Signaling
PI3K/AKT Signaling
Opioid Signaling Pathwsy
GNRH Signaling
Cellular Immune Response
Tec Kinase Signaling
Calcium Signaling
Sphingosine−1−phosphate Signaling
Hereditary Breast Cancer Signaling
GABA Receptor Signaling
Reelin Signaling in Neurons
iCOS−iCOSL Signaling in T Helper Cells
Phagosome Formation
Allograft Rejection Signaling
Macropinocytosis Signaling
NF−κB Signaling
Role of Macrophages, Fibroblasts and Endothelial Cells in Rheumatoid Arthritis
Huntington's Disease Signaling
NRF2−mediated Oxidative Stress Response
GP6 Signaling Pathway
LXR/RXR Activation
VDR/RXR Activation
Cellular Stress and Injury
Ingenuity Toxicity List Pathways
CTLA4 Signaling in Cytotoxic T Lymphocytes
Communication between Innate and Adaptive Immune Cells
TREM1 Signaling
Agranulocyte Adhesion and Diapedesis
Disease−Specific Pathways
Antioxidant Action of Vitamin C
Type II Diabetes Mellitus Signaling
PPARα/RXRα Activation
PKCθ Signaling in T Lymphocytes
Granulocyte Adhesion and Diapedesis
Role of Pattern Recognition Receptors in Recognition of Bacteria and Viruses
Clathrin−mediated Endocytosis Signaling
Primary Immunodeficiency Signaling
Role of PKR in Interferon Induction and Antiviral Response
Hepatic Fibrosis / Hepatic Stellate Cell Activation
Hepatic Cholestasis
Systemic Lupus Erythematosus Signaling
Integrin Signaling
Acute Phase Response Signaling
Cell Cycle Regulation
Cytokine Signaling
Cyclins and Cell Cycle Regulation
Role of JAK1 and JAK3 in γc Cytokine Signaling
Nuclear Receptor Signaling
Th2 Pathway
CREB Signaling in Neurons
EIF2 Signaling
IL−7 Signaling Pathway
Sumoylation Pathway
Cellular Growth, Proliferation and Development
Thrombin Signaling
Endothelin−1 Signaling
Cardiac β−adrenergic Signaling
Th1 Pathway
Cdc42 Signaling
AMPK Signaling
Regulation of eIF4 and p70S6K Signaling
Gap Junction Signaling
Human Embryonic Stem Cell Pluripotency
Cardiovascular Signaling
Role of NFAT in Cardiac Hypertrophy
Calcium−induced T Lymphocyte Apoptosis
SAPK/JNK Signaling
Apoptosis
Toll−like Receptor Signaling
Biosynthesis
Salvage Pathways of Pyrimidine Ribonucleotides
Virus Entry via Endocytic Pathways
Pathogen −Influenced Signaling
Organismal Growth and Development
